# CCDC6-RET fusion protein regulates Ras/MAPK signaling through the fusion- GRB2-SHC1 signal niche

**DOI:** 10.1073/pnas.2322359121

**Published:** 2024-05-28

**Authors:** Ting Qiu, Yichao Kong, Guifeng Wei, Kai Sun, Ruijie Wang, Yang Wang, Yiji Chen, Wenxin Wang, Yun Zhang, Caihong Jiang, Peiguo Yang, Tian Xie, Xiabin Chen

**Affiliations:** ^a^School of Pharmacy, Hangzhou Normal University, Hangzhou, Zhejiang 311121, China; ^b^Key Laboratory of Elemene Class Anti-Cancer Chinese Medicines, Hangzhou Normal University, Hangzhou, Zhejiang 311121, China; ^c^Engineering Laboratory of Development and Application of Traditional Chinese Medicines, Hangzhou Normal University, Hangzhou, Zhejiang 311121, China; ^d^Collaborative Innovation Center of Traditional Chinese Medicines of Zhejiang Province, Hangzhou Normal University, Hangzhou, Zhejiang 311121, China; ^e^School of Life Sciences, Westlake University, Hangzhou 310024, China

**Keywords:** CCDC6-RET, fusion protein, liquid–liquid phase separation, kinase, signal niche

## Abstract

Rearranged during transfection (RET) rearrangement oncoprotein-mediated Ras/MAPK signaling cascade is constitutively activated in cancers. Here, we demonstrate a unique signal niche. The niche is a ternary complex based on the chimeric RET liquid–liquid phase separation. The complex comprises the rearranged kinase (RET fusion); the adaptor (GRB2), and the effector (SHC1). Together, they orchestrate the Ras/MAPK signal cascade, which is dependent on tyrosine kinase. CCDC6-RET fusion undergoes LLPS requiring its kinase domain and its fusion partner. The CCDC6-RET fusion LLPS promotes the autophosphorylation of RET fusion, with enhanced kinase activity, which is necessary for the formation of the signaling niche. Within the signal niche, the interactions among the constituent components are reinforced, and the signal transduction efficiency is amplified. The specific RET fusion–related signal niche elucidates the mechanism of the constitutive activation of the Ras/MAPK signaling pathway. Beyond just focusing on RET fusion itself, exploration of the ternary complex potentially unveils a promising avenue for devising therapeutic strategies aimed at treating RET fusion–driven diseases.

## Significance

The generation of fusion kinases through gene rearrangement represents a primary mechanism of rearranged during transfection (RET) oncogenic conversion. The change of location, increased expression, and constitutive activation lead to aberrant cell proliferation and carcinogenesis. Our research has unveiled the inherent capability of CCDC6-RET to undergo liquid–liquid phase separation (LLPS), highlighting the interdependence between LLPS and kinase activity. The presence of RET fusion protein leads to the constitutive activation of the Ras/MAPK signaling pathway. Within this context, we have identified a specific signal niche, which is a chimeric RET LLPS-based ternary complex. This complex comprises of three key components: the rearranged kinase known as RET fusion, the adaptor protein GRB2; and the effector protein SHC1. Together, they orchestrate the Ras/MAPK signal cascade, which is dependent on tyrosine kinase.

The proto-oncogene rearranged during transfection (RET) encodes a transmembrane receptor tyrosine kinase, which consists of three main parts: glycosylated extracellular domain, single-pass transmembrane domain, and intracellular tyrosine kinase domain (1–3). Upon ligand binding, such as glial cell-line derived neurotrophic factor (GDNF), and other associated proteins to the extracellular domain, the intracellular tyrosine kinase domain undergoes dimerization. This is followed by the autophosphorylation of critical tyrosines. Subsequently, this activates downstream signaling pathways, including the Ras/MAPK, PI3K/AKT pathways (4–9). RET is involved in various physiological and developmental functions, such as cell proliferation, migration, apoptosis, and neural and neuroendocrine tissue development (10). However, aberrant RET, in particular somatic point mutations and cytogenetic rearrangement, can turn on the oncogenic potential of RET and generate constitutive kinase activation without ligand binding, leading to the development of various cancers, including familial medullary thyroid carcinomas, multiple endocrine neoplasia type 2A and 2B (MEN2A and MEN2B) (11–13), Hirschsprung disease (14), and papillary thyroid cancer (15–17).

Genomic rearrangement is one primary mechanism of RET oncogenic conversion (18, 19). The most common breakpoint occurs in intron 11, leading to the fusion with the only cytoplasmic kinase domain, changing the cellular location of RET protein from membrane to cytoplasm. The fusion partner usually contains the coiled-coil domain, which contributes to the dimerization domains of the fusion proteins, resulting in ligand-independent kinase activity (20, 21). Otherwise, due to substituting the transcriptional promotor with the fusion partner, the expression of RET proto-oncogene increases (22, 23). The change of location, increased expression, and constitutive activation lead to aberrant cell proliferation and carcinogenesis. More than 35 RET-related fusions have been reported; CCDC6-RET was the first to be identified and present in various cancers, particularly, papillary thyroid cancer (PTC) and non–small cell lung adenocarcinoma (NSCLC). It was becoming an important molecular marker for pathology detection as the high-frequency detection rate (24). Coiled-coil domain containing 6 (CCDC6) is ubiquitously expressed across a variety of tissues. It plays a role in numerous cellular processes and has been linked to certain cancers (25, 26). Notably, the CCDC6-RET fusion gene is predominantly observed in papillary thyroid cancer and lung cancer. Moreover, the CCDC6-RET fusion protein exhibits a distinct response to traditional RET inhibitors. This response is different when compared to other prevalent RET fusion proteins, such as KIF5B-RET and NCOA4-RET (27–34), suggesting a specific mechanism of CCDC6-RET in tumor development. However, its function and mechanism in cancer were not well understood.

Amino acid composition and disordered region analysis suggested the intrinsic protein disordered regions (IDRs) in CCDC6-RET, which may mediate multivalent interactions and promote phase separation (35, 36). Due to rearrangement, the fusion protein exhibited altered cellular localization and abnormal kinase activity. The presence of IDRs in the partner suggested that CCDC6-RET may undergo LLPS. And, it has been described that CCDC6-RET can form protein granules with interacting proteins to activate Ras/MAPK signaling (37). Biology condensate formed through LLPS is a novel mechanism for kinase regulation of signaling pathways and can serve as a reaction platform for kinases (38). Yet there is a lack of understanding of the oncogenic mechanisms and activation modes of CCDC6-RET fusion. Indeed, the role of partner proteins for fusion proteins is also unknown. Therefore, an intensive study of the specific role of CCDC6-RET in related cancers will be an important guide to broaden the mechanism of RET-driven tumorigenesis and develop targeted therapies for CCDC6-RET-positive tumors.

In this study, we demonstrated that CCDC6-RET fusion protein is able to undergo LLPS in the cytoplasm without ligands or any other associated proteins, athough very few fusion protein granules were observed under normal conditions. The fusion partner, CCDC6-101aa with IDRs, shows a similar ability to undergo LLPS. Such ability of fusion partners to undergo LLPS is essential for the LLPS and activation of CCDC6-RET. The extent of phase separation varies among different fusion proteins, mainly determined by the characteristics of partners. The phase separation of CCDC6-RET also relies on the intact enzymatic activity of the kinase domain. On the CCDC6-RET LLPS, we uncovered that CCDC6-RET recruited GRB2 and SHC1 simultaneously to form a membraneless signaling niche to transduce the Ras/MAPK signaling. The membraneless ternary complexes are established through phase separation facilitated by CCDC6-RET. Within the signal niche, the interactions among the constituent components are reinforced, particularly the potency between CCDC6-RET and GRB2. The elucidation of the distinctive CCDC6-RET LLPS-dependent signal niche unveils the mechanism underpinning the constitutive activation of the Ras/MAPK signaling pathway. The endeavor to target RET fusion proteins has proven to be a challenging pursuit. The revelation of the ternary complex introduces a broad therapeutic avenue for managing RET fusion–driven diseases.

## Results

### The Partner and RET Kinase Domain Form Fusion Protein Condensate via Liquid–liquid Phase Separation.

**Fig. 1.**
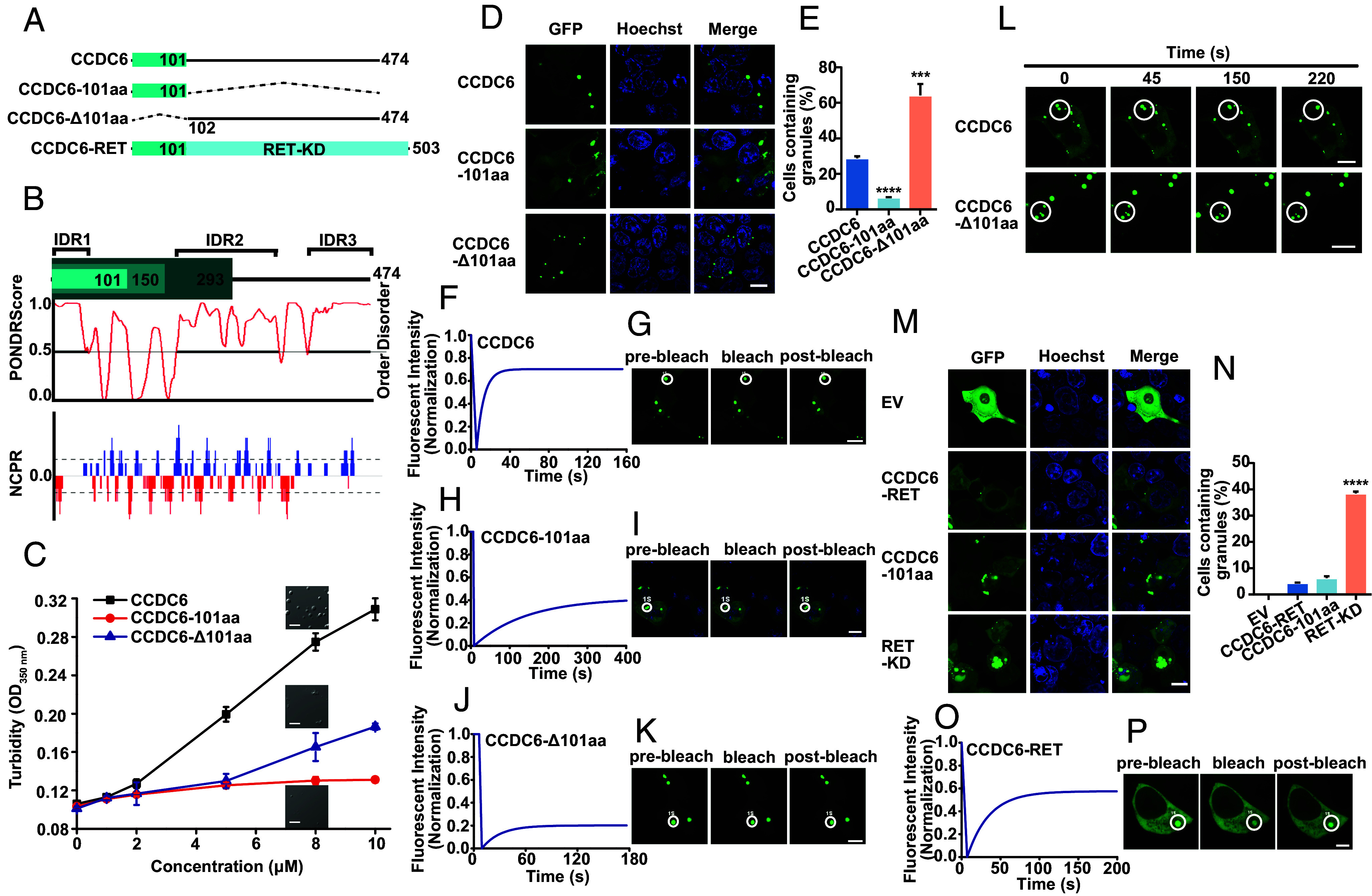
Both CCDC6 and CCDC6-RET fusion protein form condensates via liquid–liquid phase separation. (*A*) Schematic of CCDC6, CCDC6-101aa, CCDC6-Δ101aa, and its portion fused to RET, named CCDC6-RET. RET-KD: RET kinase domain. (*B*) CCDC6 (full length) amino acids aligned with PONDR (predictor of natural disordered regions) and NCPR (net charge per residue) analysis. IDR: intrinsically disordered regions. (*C*) The turbidity assays of purified recombinant CCDC6, CCDC6-101aa, and CCDC6-Δ101aa proteins were measured by concentrations from 0 to 10 µM at OD350 nm in 5% PEG 8000. The phase separation behaviors were shown at a concentration of 8 µM. Error bars indicate SD of n = 3 biologically independent samples. (Scale bar, 5 µm.) (*D*) Live-cell imaging of EGFP-tagged CCDC6, CCDC6-101aa, and CCDC6-Δ101aa expressed in HEK293T cells, respectively, n ≥ 3. (Scale bar, 10 µm.) (*E*) Proportion of cells with protein condensates upon transfection of CCDC6, CCDC6-101aa, and CCDC6-Δ101aa in HEK293T cells. Error bars indicate SD from three regions in the imaged sample selected for counting, with each region containing more than 200 total cells. ****P < 0.0001 and ***P < 0.001 versus CCDC6 by Student’s *t* test. (*F* and *G*) FRAP of CCDC6-EGFP transfected in HEK293T cells. The FRAP curves are shown in *F* and the corresponding droplet at the bleached area in *G*. (Scale bar, 10 µm.) (*H* and *I*) FRAP of CCDC6-101aa-EGFP transfected in HEK293T cells. The FRAP curves in *H* and the corresponding droplet at the bleached area in *I*. (Scale bar, 10 µm.) (*J* and *K*) FRAP of CCDC6-Δ101aa-EGFP transfected in HEK293T cells. The FRAP curves are shown in *J* and the corresponding droplet at the bleached area in *K*. (Scale bar, 10 µm.) (*L*) Time-lapse imaging of EGFP-tagged CCDC6 and CCDC6-Δ101aa droplet fusion expressed in HEK293T cells. (Scale bar, 10 μm.) See also Movie S1. (*M*) Live-cell imaging of EV and EGFP-tagged CCDC6-RET, CCDC6-101aa, and RET-KD expressed in HEK293T cells, respectively, n ≥ 3. (Scale bar, 10 µm.) (*N*) Proportion of cells with protein condensates upon transfection of EV and EGFP-tagged CCDC6-RET, CCDC6-101aa, and RET-KD in HEK293T cells. Error bars indicate SD from three regions in the imaged sample selected for counting, with each region containing more than 200 total cellswith. ****P < 0.0001 versus CCDC6-RET by Student’s *t* test. (*O* and *P*) FRAP of CCDC6-RET-EGFP transfected in HEK293T cells. The FRAP curves are shown in *O* and the corresponding droplet at the bleached area in *P*. (Scale bar, 10 µm.)

The CCDC6-RET fusion protein contains two parts: the kinase domain of RET in the C terminus and the partner containing the first 101 amino acids of CCDC6 (CCDC6-101aa) (Fig. 1*A*), which features long coiled-coil domains with three intrinsic disorder regions (IDRs) (Fig. 1*B*). Natural disordered regions are intrinsic drivers of liquid–liquid phase separation of many proteins (39). Thus, CCDC6-101aa, which contains the first IDR of CCDC6, has the potential to undergo LLPS not only itself but also its fusion protein.

To investigate whether CCDC6 protein undergoes liquid–liquid phase separation like other disorder proteins, we expressed the full length (FL) of CCDC6 in *E. coli*. CCDC6 exhibits concentration-dependent LLPS upon adding the molecular crowding agent PEG 8000 (*SI Appendix*, Fig. S1 *A* and *B*). Given that the IDR1 of CCDC6 was in the first exon, we examined its role in phase separation. We generated constructs in which the first exon (the 101aa) was expressed alone (101aa), and the 101aa was deleted (Δ101aa). We found that CCDC6-101aa was able to LLPS and dispensable for LLPS of CCDC6. The turbidity of the solution containing 5% PEG 8000 at a wavelength of 350 nm quantitatively demonstrated the concentration-dependent effect of CCDC6 for LLPS, while CCDC6-101aa has little such effect. The absorbance of CCDC6-Δ101aa was smaller than CCDC6 at the same concentration but still exhibited a tendency to increase with concentration (Fig. 1*C*). Real-time imaging revealed that the CCDC6 formed larger sphere condensates, while smaller condensates were formed by CCDC6-101aa in the solution (Fig. 1*C* and *SI Appendix*, Fig. S1*G*). Deletion of 101aa slightly promoted LLPS at lower concentrations, and the condensates formed trended to show irregular agglomeration (Fig. 1*C* and *SI Appendix*, Fig. S1 *C*−*G*). These results suggest that CCDC6-101aa is sufficient for LLPS, albeit weaker than FL CCDC6, and may regulate the solubility of FL CCDC6.

We next assessed the protein phase separation in cells. In line with their behavior in vitro, few droplets were formed for IDR1 containing 101aa fragment, while FL CCDC6 and CCDC6-Δ101aa formed more LLPS droplets (Fig. 1 *D* and *E* and *SI Appendix*, Fig. S1 *H* and *I*). To determine the dynamics of these condensates in cells, we performed fluorescence recovery after photobleaching (FRAP) analysis of GFP-tagged CCDC6 (FL, 101aa or Δ101aa). We observed that the FL CCDC6 showed a faster recovery than the other two proteins (Fig. 1 *F*−*K*). Furthermore, real-time imaging demonstrated that over time, the FL CCDC6 condensates fused with each other to enlarge their size, while CCDC6-Δ101aa condensates did not fuse regardless of distance in the same time window (Fig. 1*L* and Movie S1). Therefore, these results indicate that the first exon of CCDC6 (101aa) plays a critical role in the dynamic of CCDC6 condensates.

To identify the role of 101aa IDR in condensate formation, we expressed the CCDC6-RET fusion protein and the RET kinase domain (RET-KD) in cells. Few fusion proteins undergo LLPS, while RET-KD tended to form irregular aggregates (Fig. 1 *M* and *N* and *SI Appendix*, Fig. S1 *J* and *K*). FRAP analysis showed minimal recovery of the RET-KD aggregate and a rapid, high recovery rate of RET fusion proteins (Fig. 1 *O* and *P* and *SI Appendix*, Fig. S1 *L* and *M*). Collectively, these results suggest that the CCDC6-101aa is sufficient to drive LLPS and regulate the material property of the CCDC6-RET fusion protein.

### Like the RET Oncogenic Activity, CCDC6-RET Fusion Participates in the Ras/MAPK Signal Pathway.

**Fig. 2.**
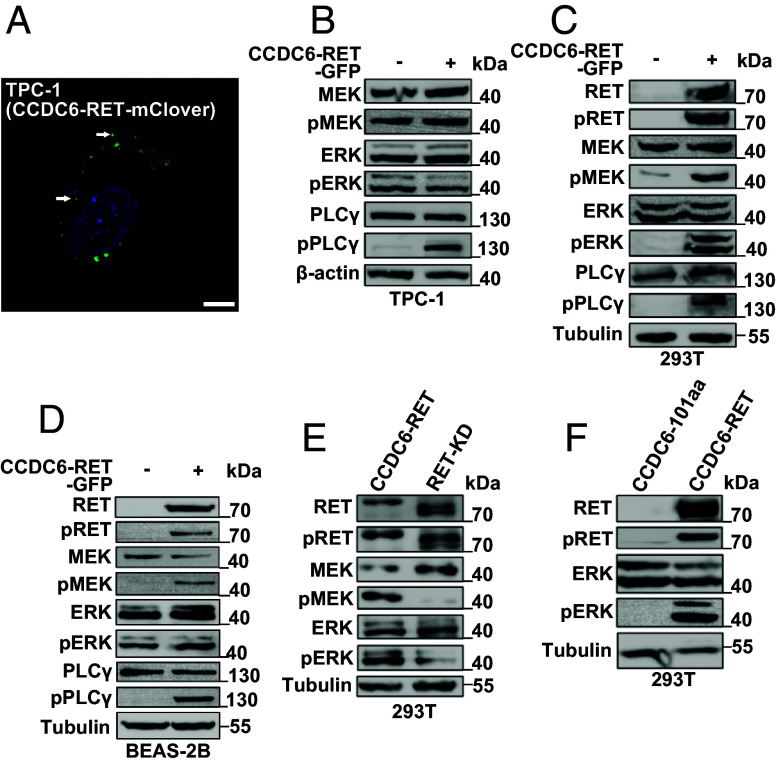
CCDC6-RET fusion drives the Ras/MAPK signal pathway. (*A*) Live-cell imaging of TPC-1 cells with endogenous mClover-tagging of CCDC6-RET. (Scale bar, 10 μm.) (*B*) Western blotting upon expression of EV or CCDC6-RET-EGFP in TPC-1 cells. (*C*) Western blotting upon expression of EV or CCDC6-RET-EGFP in HEK293T cells. (*D*) Western blotting upon expression of EV or CCDC6-RET-EGFP in BEAS-2B cells. (*E*) Western blotting upon expression of CCDC6-RET-EGFP or RET-KD-EGFP in HEK293T cells. (*F*) Western blotting upon expression of CCDC6-101aa-EGFP or CCDC6-RET-EGFP in HEK293T cells.

RET, a membrane-bound receptor, participates in the oncogenic Ras/MAPK and PI3K/AKT signal pathways in various cancers. In neurons, the kinase was activated by its ligands and coreceptors (40). In contrast, the fusion protein CCDC6-RET alters its localization and leads to constitutive activation in a ligand-independent manner (41). First, we verified that the CCDC6-RET localized in the cytoplasm, not at the membrane, and formed LLPS condensates by knockin the mClover fluorescent tag following CCDC6-RET in the patient-derived cancer cell TPC-1 (thyroid papillary carcinoma) (Fig. 2*A* and *SI Appendix*, Fig. S2*C*). TPC-1 cells harboring CCDC6-RET fusion protein exhibited activated Ras/MAPK signaling and weak PI3K/AKT signaling (Fig. 2*B* and *SI Appendix*, Fig. S2 *A* and *B*). The Ras/MAPK signal was also activated in other cell lines transfected with CCDC6-RET (Fig. 2 *C* and *D* and *SI Appendix*, Fig. S2*D*). This activation was independent of the tag’s placement at either the N or C terminus, as well as the specific types involved (*SI Appendix*, Fig. S2 *E* and *F*). The same results were also exhibited in the CCDC6-RET-stable cells (*SI Appendix*, Fig. S2*G*). Neither CCDC6 (FL or 101aa) nor the RET kinase domain alone affected the signal pathway (Fig. 2 *E* and *F*).

Gene transcription and protein expression of CCDC6-RET were low in TPC-1 cells (Fig. 2*A* and *SI Appendix*, Fig. S2 *A*–*C*). Upon transfection with exogenous CCDC6-RET, PI3K/AKT signaling was enhanced, while the already activated Ras/MAPK signaling was not further augmented (Fig. 2*B*). These results suggest that the role of the CCDC6-RET fusion protein in the Ras/MAPK signaling pathway is a critical factor in the carcinogenesis of thyroid papillary cancer.

### The Ability to Undergo LLPS of CCDC6-101aa Is Essential for the LLPS and Activation of the CCDC6-RET Fusion Protein.

**Fig. 3.**
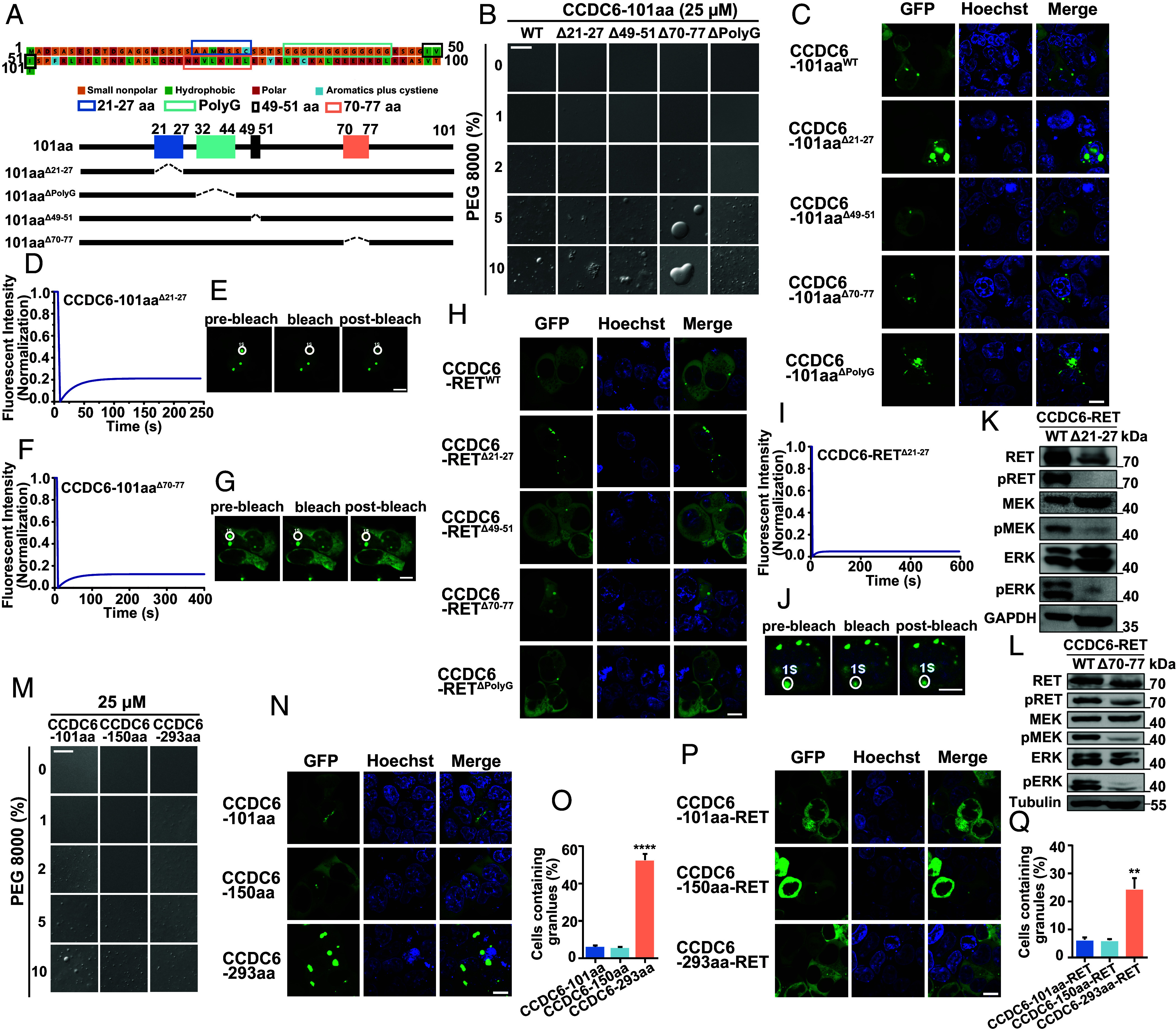
The intact IDRs of the partner are prerequisite for both LLPS and activation of the fusion protein. (*A*) The amino acid sequence of CCDC6-101aa is shown with major segments marked colors. (*B*) Comparison of the phase separation behaviors between purified recombinant mutant forms of CCDC6-101aa, including WT (wild type), Δ21-27aa, Δ49-51aa, Δ70-77aa, and ΔPloyG, with increasing concentration of PEG 8000. See also *SI Appendix*, Figs. S1 and S3. (Scale bar, 20 µm.) (*C*) Live-cell imaging of various CCDC6-101aa-EGFP mutations, including WT, Δ21-27aa, Δ49-51aa, Δ70-77aa, and ΔPloyG expressed in HEK293T, respectively, n ≥ 3. (Scale bar, 10 µm.) (*D* and *E*) FRAP of CCDC6-101aa^Δ21-27^-EGFP transfected in HEK293T cells. The FRAP curves are shown in *D* and the corresponding droplet at the bleached area in *E*. (Scale bar, 10 µm.) (*F* and *G*) FRAP of CCDC6-101aa^Δ70-77^-EGFP transfected in HEK293T cells. The FRAP curves are shown in *F* and the corresponding droplet at the bleached area in *G*. (Scale bar, 10 µm.) (*H*) Live-cell imaging of various CCDC6-RET-EGFP mutations, including WT, Δ21-27aa, Δ49-51aa, Δ70-77aa, and ΔPloyG expressed in HEK293T, respectively, n ≥ 3. (Scale bar, 10 µm.) (*I* and *J*) FRAP of CCDC6-RET^Δ21-27^-EGFP transfected in HEK293T cells. The FRAP curves are shown in *I* and the corresponding droplet at the bleached area in *J*. (Scale bar, 10 µm.) (*K*) Western blotting upon expression of EGFP-tagged CCDC6-RET or CCDC6-RET^Δ21-27^ in HEK293T cells. (*L*) Western blotting upon expression of EGFP-tagged CCDC6-RET or CCDC6-RET^Δ70-77^ in HEK293T cells. (*M*) Comparison of the phase separation behaviors between purified recombinant protein, containing CCDC6-101aa, CCDC6-150aa, and CCDC6-293aa, with increasing concentration of PEG 8000. See also *SI Appendix*, Fig. S3. (Scale bar, 20 µm.) (*N*) Live-cell imaging of CCDC6-101aa, CCDC6-150aa, and CCDC6-293aa expressed in HEK293T cells, respectively, n ≥ 3. (Scale bar, 10 µm.) (*O*) Proportion of cells with protein condensates upon transfection of CCDC6-101aa, CCDC6-150aa, and CCDC6-293aa in HEK293T cells. Error bars indicate SD from three regions in the imaged sample selected for counting, with each region containing more than 200 total cells. ****P < 0.0001 versus CCDC6-101aa by Student’s *t* test. (*P*) Live-cell imaging CCDC6-101aa-RET, CCDC6-150aa-RET, and CCDC6-293aa-RET expressed in HEK293T cells, respectively, n ≥ 3. (Scale bar, 10 µm.) (*Q*) Proportion of cells with protein condensates upon transfection of CCDC6-101aa-RET, CCDC6-150aa-RET, and CCDC6-293aa-RET in HEK293T cells. Error bars indicate SD from three regions in the imaged sample selected for counting, with each region containing more than 200 total cells. **P < 0.01 versus CCDC6-101aa-RET by Student’s *t* test.

To investigate the involvement of CCDC6-101aa in the LLPS of CCDC6-RET fusion protein, we analyzed the amino acid composition of CCDC6-101aa. It contains a few polar amino acids and many small nonpolar amino acids, such as glycine. Proteins with specific sequences that include low-complexity, intrinsically disordered, or prion-like domains are more likely to undergo LLPS. These domains often have amino acids like glycine, serine, and glutamine, which favor phase separation. Aromatic amino acids usually stabilize the condensed phase. Polar amino acids contribute to LLPS by forming electrostatic interactions and hydrogen bonds. These interactions help attract proteins and form condensates (42–45). To identify the key regions for LLPS, we deleted four specific regions: the glycine-rich region (PolyG), the region with all four types of amino acids (21-27aa), the hydrophobic amino acid region (49-51aa), and the hydrophobic-polar-rich region (70-77aa), respectively (Fig. 3*A*). The unique amino acid compositions of these regions suggest a possible role in initiating LLPS or protein–protein interactions. Deletion of the PolyG or 49-51aa led to smaller droplets compared to the full length of CCDC6-101aa. However, deletion of 70-77aa, the hydrophobic-polar-rich region, resulted in droplets that tend to aggregate into irregular and larger condensates. In contrast, deletion of 21-27aa produced small droplets prone to aggregation (Fig. 3*B* and *SI Appendix*, Fig. S3 *A*–*H*). We then assessed the droplet formation of CCDC6-101aa deletion mutants in 293T cells (Fig. 3*C*). Consistent with their behavior in vitro, deletion of 21-27aa led to irregular condensates, while the deletion of the 70-77aa region formed circular condensates, both with low mobility (Fig. 3 *C*–*G*). These findings suggest that the intact CCDC6-101aa is essential for LLPS.

To explore the functional relevance of CCDC6-101aa in CCDC6-RET fusion protein, we introduced the aforementioned deletions into the CCDC6-RET fusion construct. Consistent with the deletion mutants of CCDC6-101aa, deletion 21-27aa or 70-77aa in CCDC6-RET resulted in larger condensates with low recovery rate after bleaching (Fig. 3 *H*–*J*). Subsequently, the activation of cytosolic Ras/MAPK signaling was examined. Compared to wild-type CCDC6-RET, the Δ70-77 mutant reduced the level of activated cytosolic Ras/MAPK signaling, while the Δ21-27 mutant lost the capability of productive Ras/MAPK signaling (Fig. 3 *K* and *L* and *SI Appendix*, Fig. S3 *M* and *N*). These data demonstrate that the CCDC6-101aa is crucial not only for the LLPS but also for activating the CCDC6-RET fusion protein. In other words, the natural LLPS of CCDC6 101aa is indispensable for the function of CCDC6-RET.

To further explore the role of the N-terminal of CCDC6-RET in the formation of condensates, we constructed two additional naturally occurring rearrangements of CCDC6 and RET, CCDC6-150aa-RET and CCDC6-293aa-RET (26, 46). Following a similar approach employed for studying CCDC6-101aa-RET, we initially constructed the expression system for CCDC6-150aa and CCDC6-293aa. CCDC6-293aa promoted LLPS at lower threshold concentrations in vitro, while the proteins tend to form larger and numerous condensates in cells. The LLPS behavior of CCDC6-150aa resembled that of CCDC6-101aa, both in vitro and in vivo (Fig. 3 *M*–*O* and *SI Appendix*, Fig. S3 *I*–*L*). This discrepancy may be attributed to the presence of two IDRs in CCDC6-293aa (*SI Appendix*, Fig. S3 *O* and *P*). Moreover, the distinction in phase separation patterns between CCDC6-150aa and CCDC6-293aa extends to their respective fusion proteins, CCDC6-150aa-RET and CCDC6-293aa-RET. CCDC6-150aa-RET exhibited a similar number of granules as CCDC6-101aa-RET, whereas CCDC6-293aa-RET showed a significantly increased number of granules compared to the other two fusions (Fig. 3 *P* and *Q*). These results indicate the significant role of the N-terminal region in the occurrence of CCDC6-RET phase separation. We conducted further research to determine whether the naturally occurring RET fusion proteins, KIF5B-RET and NCOA4-RET, are capable of undergoing LLPS. Both KIF5B-RET and NCOA4-RET form protein foci. Notably, the variations in the N-terminal domains of fusion proteins contribute to the nonidentity of the protein foci generated by KIF5B-RET and NCOA4-RET in comparison to those formed by CCDC6-RET (*SI Appendix*, Fig. S3*Q*).

### The LLPS of Fusion Protein Is Dependent on the RET Kinase Activity and Strengthens the Kinase Function.

**Fig. 4.**
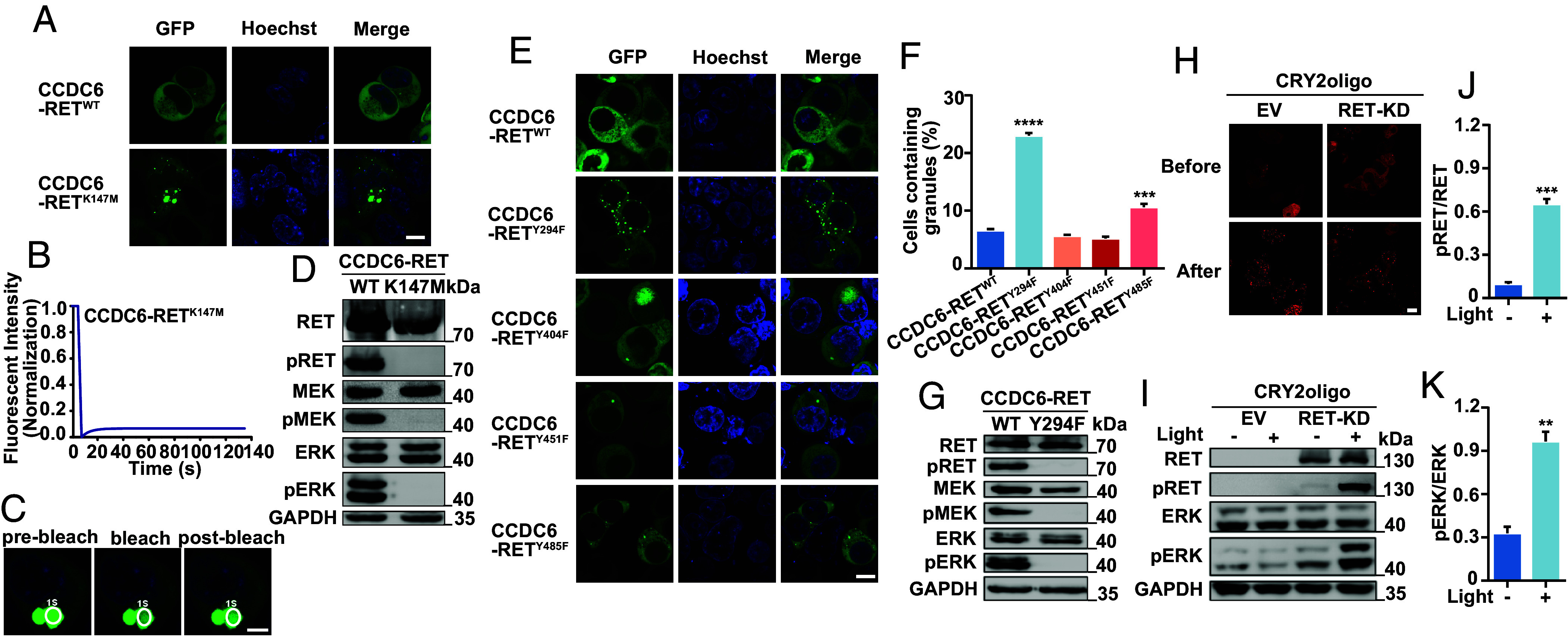
The LLPS interplay with RET kinase activity and function. (*A*) Live-cell imaging of CCDC6-RET-EGFP or CCDC6-RET^K147M^-EGFP mutant expressed in HEK293T cells, respectively, n ≥ 3. (Scale bar, 10 µm.) (*B* and *C*) FRAP of CCDC6-RET^K147M^-EGFP transfected in HEK293T cells. The FRAP curves in *B* and the corresponding droplet at the bleached area in *C*. (Scale bar, 5 µm.) (*D*) Western blotting upon expression of CCDC6-RET-EGFP or CCDC6-RET^K147M^-EGFP mutant in HEK293T cells. (*E*) Live-cell imaging of CCDC6-RET-EGFP or its related phosphorylation site mutations consisting of Y294F, Y404F, Y451F, and Y485F expressed in HEK293T cells, n ≥ 3. (Scale bar, 10 µm.) (*F*) Proportion of cells with protein condensates upon transfection of CCDC6-RET-EGFP, Y294F, Y404F, Y451F, or Y485F mutations in HEK293T cells. Error bars indicate SD from three regions in the imaged sample selected for counting, with each region containing more than 200 total cells. ****P < 0.0001 and ***P < 0.001 versus CCDC6-RET by Student’s *t* test. (*G*) Western blotting upon expression of CCDC6-RET-EGFP or CCDC6-RET^Y294F^ -EGFP mutant in HEK293T cells. (*H*–*K*) The RET-fusion active Ras/MAPK via LLPS. The time-lapse images of RET-KD linked to CRY2oligo-mCherrry before/after 488 nm blue light induced in *H*. Western blot upon expression 24 h of EV or RET-KD linked to CRY2oligo-mCherrry illuminated by 488 nm blue light at 20% power for 5 min/cycle remaining three cycles in 96-well plates in *I*, the analyses of pRET values in *J* and pERK in *K*. CRY2oligo-mCherrry alone was used as a control, n ≥ 3. For all panels, error bars represent ± SEM, representative of n ≥ 3 independent experiments. **P < 0.01 and ***P < 0.001 versus before light-induced by Student’s *t* test. (Scale bar, 10 µm.)

The K758M mutation in RET protein has been previously characterized as a kinase-deficient variant (35). In this study, we constructed the corresponding mutant, K147M, in CCDC6-RET. The K147M mutant of CCDC6-RET can still form cytoplasmic foci with irregular shapes (Fig. 4*A*). The small foci were dynamic while rapidly aggregating into irregular condensate. Notably, the larger and irregular condensate displayed little recovery after bleaching (Fig. 4 *B* and *C*). Consistent with the RET K758M with no tyrosine kinase activity, the CCDC6-RET K147M mutant almost eliminated the cytosolic Ras/MAPK signaling. Meanwhile, we did not detect the autophosphorylation of CCDC6-RET K147M (Fig. 4*D*).

The activation of RET tyrosine kinase relies on the phosphorylation of specific tyrosine residues. The membrane-located RET protein was activated after autophosphorylation on the intracellular tyrosine residues following the ligand-induced extracellular dimerization (8). The formation of fusion proteins leads to a relocation of the protein from the membrane to the cytoplasm, resulting in the autophosphorylation of multiple tyrosine residues without ligands. To identify whether the phosphorylation is critical for the stable LLPS of CCDC6-RET, we constructed four single mutants with essential phosphorylation sites Y294, Y404, Y451, and Y485 (corresponding to Y905, Y1015, Y1062, and Y1096 in full length of RET) mutated to phenylalanine (F). These mutations also have been identified in different cancer cells. Among these tyrosine residue mutants, the CCDC6-RET Y294F mutant lost enzymatic activity but formed more spherical and dynamic condensates (Fig. 4 *E*–*G* and *SI Appendix*, Fig. S4 *A* and *B*). The residue Y905 (corresponding to the Y294 in CCDC6-RET) located in the activation loop of RET is critical for RET signaling in cells. The mutation Y905F disrupts ATP binding to the active loop (8, 9). It is reasonable to assume that the corresponding mutation similarly disturbed ATP binding to the RET fusion proteins. Other tyrosine residues mutants showed minor changes in the LLPS of CCDC6-RET, while displaying different effects on the RET kinase activity (Fig. 4 *E* and *F* and *SI Appendix*, Fig. S4 *C* and *D*). Meanwhile, as the number of mutated phosphorylation sites increased, the number of condensates decreased (*SI Appendix*, Fig. S4 *E* and *F*). Loss of RET activity results in the formation of larger condensate with low recovery (*SI Appendix*, Fig. S4 *E*–*I*), indicating that the phosphorylated tyrosines play an important role in the formation of RET fusion protein condensates. Other mutants detected in cancers such as L790F, V804M, and M918T (corresponding to the L179F, V193M, and M307T in CCDC6-RET), which result in resistance to different inhibitors, had diverse effects on the phase separation but did not eliminate the kinase activity (*SI Appendix*, Fig. S4 *J*–*M*).

Furthermore, to gain further insights into the reciprocal regulation of condensate formation and kinase activity, we used the optoCRY2oligo system. The Arabidopsis photoreceptor cryptochrome 2 (CRY2) distributed homogeneously in cells in darkness (36). However, CRY2 oligomerized reversibly upon blue light illumination through their PHR domains (47). By fusing the CRY2 to RET-KD, we were able to control the formation of kinase-mediated LLPS. Due to the fusion to CRY2, the RET-KD distributed homogeneously in the cytoplasm initially, and numerous CRY2-RET-KD LLPS condensates were formed after 488 nm-light-induction within one second. Meanwhile, the RET signaling pathway was activated with the increased phosphorylation of RET and ERK (Fig. 4 *H*–*K*). These results suggest that the formation of condensates and kinase activity are regulated reciprocally. The normal kinase function is essential for the dynamic behavior of CCDC6-RET condensates, while the liquidity of condensates enhances the kinase activity.

### The Adaptor and Effector Proteins Facilitate the LLPS of CCDC6-RET.

**Fig. 5.**
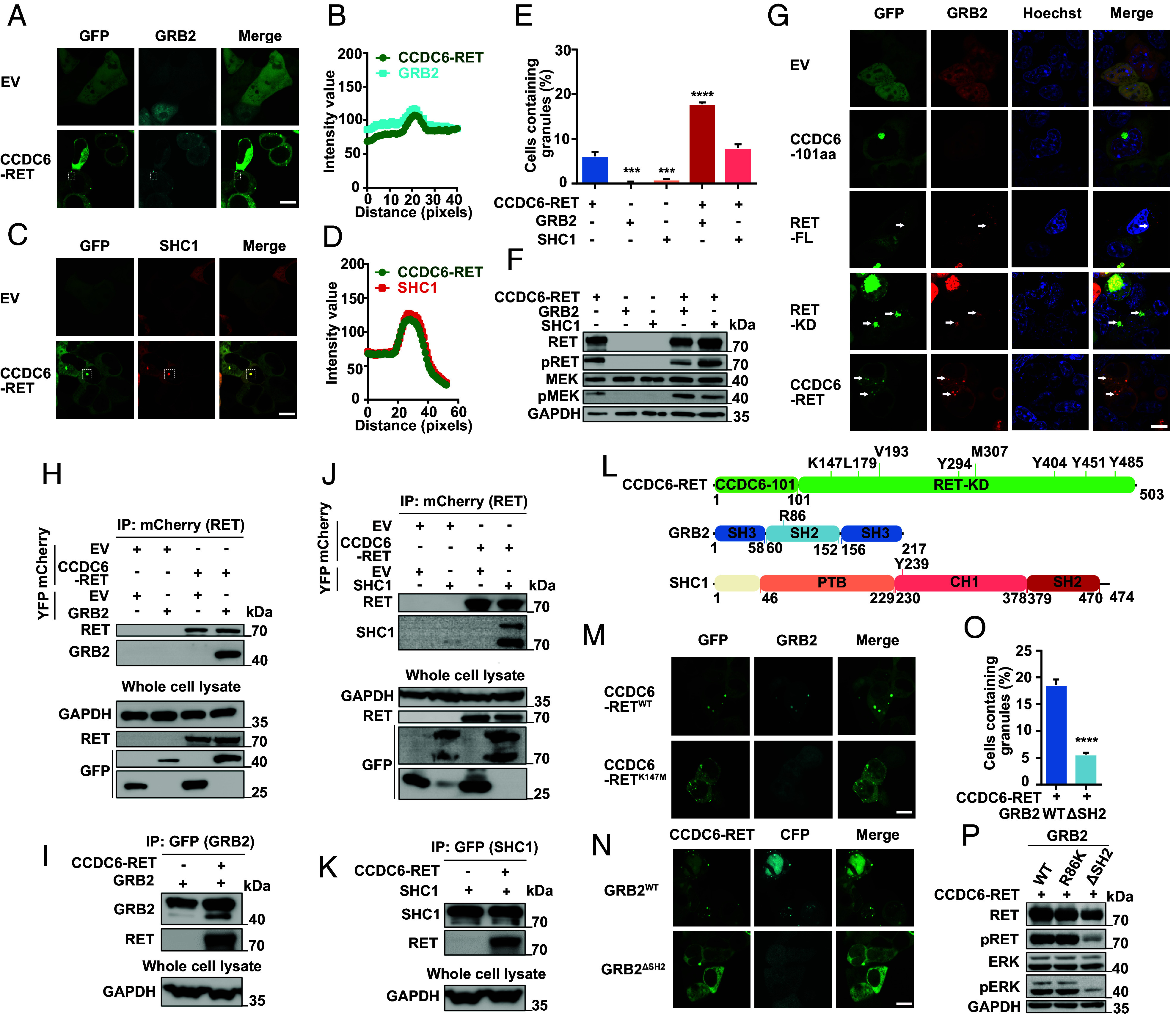
The interacting proteins of RET are recruited by fusion protein to form granules. (*A* and *B*) Live-cell imaging of HEK293T cells with cotransfected CCDC6-RET-EGFP and GRB2-ECFP in *A* and the plot of the colocalization of CCDC6-RET with GRB2 in *B*, the analyzed foci is within the dashed square, n ≥ 3. (Scale bar, 10 µm.) (*C* and *D*) Live-cell imaging of HEK293T cells with cotransfected CCDC6-RET-EGFP and SHC1-mCherry in *C* and the plot of the colocalization of CCDC6-RET with SHC1 in *D*, the analyzed foci is within the dashed square, n ≥ 3. (Scale bar, 10 µm.) (*E*) Proportion of cells with protein granules upon separate transfection of CCDC6-RET-EGFP, GRB2-ECFP, and SHC1-mCherry or cotransfection CCDC6-RET-EGFP with GRB2-ECFP or SHC1-mCherry. Error bars indicate SD from three regions in the imaged sample selected for counting, with each region containing more than 200 total cells. ****P < 0.0001 and ***P < 0.001 versus CCDC6-RET by Student’s *t* test. (*F*) Western blotting upon separate transfection of CCDC6-RET-EGFP, GRB2-ECFP, and SHC1-mCherry and cotransfection CCDC6-RET-EGFP with GRB2-ECFP or SHC1-mCherry in HEK293T cells. (*G*) Live-cell imaging of GRB2-mCherry expressed in HEK293T cells with EGFP and EGFP tagging of C6-101aa, RET-FL (the full length), RET-KD, and CCDC6-RET. The white arrows indicate representative GRB2 protein granules with local enrichment of EGFP-tagged protein granules (multiple nonhighlighted granules also show colocalization), n ≥ 3. (Scale bar, 10 µm.) (*H*) Anti-mCherry CO-IP of EYFP-tagged GRB2 transfected in HEK293T cells with coexpression EV or mCherry-tagged CCDC6-RET, respectively, followed by western blotting. (*I*) Anti-GFP CO-IP of EYFP-tagged GRB2 transfected in HEK293T cells with coexpression EV or mCherry-tagged CCDC6-RET, respectively, followed by western blotting. (*J*) Anti-mCherry CO-IP of EYFP-tagged SHC1 transfected in HEK293T cells with co-expression EV or mCherry-tagged CCDC6-RET, respectively, followed by western blotting. (*K*) Anti-GFP CO-IP of EYFP-tagged SHC1 transfected in HEK293T cells with co-expression EV or mCherry-tagged CCDC6-RET, respectively, followed by western blotting.(*L*) Domain structure schematic of CCDC6-RET, GRB2, and SHC1. SH3: SH3 domain; SH2: SH2 domain; PTB: PTB domain; CH1: CH1 domain. (*M*) Live-cell imaging of HEK293T cells with cotransfected EGFP-tagged CCDC6-RET or CCDC6-RETK147M mutant and GRB2-ECFP, n ≥ 3. (Scale bar, 10 µm.) (*N*–*P*) The SH2 domain of GRB2 is consequence for the formation of droplets and the Ras/MAPK activity. Live-cell imaging of HEK293T cells with cotransfected ECFP-tagged GRB2 or ECFP-tagged GRB2^ΔSH2^ mutant and CCDC6-RET-EGFP in *N*, the proportion of cells with protein granules in *O* and western blotting in *P*, n ≥ 3. Error bars indicate SD from three regions in the imaged sample selected for counting, with each region containing more than 200 total cells, ****P < 0.0001 versus GRB2^WT^ by Student’s *t* test. (Scale bar, 10 µm.)

The autophosphorylation of various tyrosine residues in RET serves as docking sites for downstream adaptors, such as SHC1, GRB2, and STAT3, leading to the activation of multiple signaling pathways (7, 48). We and other researchers demonstrated that CCDC6-RET activates these pathways similarly to RET (49, 50) (Fig. 2). Tulpule et al. proposed that CCDC6-RET forms membraneless protein granules in the cytoplasm to mediate Ras/MAPK signaling through the colocalization with downstream signaling proteins, such as GRB2, SHC1, SHP2, SOS1, etc. (37), and we also obtained the similar phenomenon (Fig. 5 *A*–*D*). At the same time, we found that the number of granules containing both CCDC6-RET and GRB2 or CCDC6-RET and SHC1 was more abundant in comparison to those containing CCDC6-RET alone, while very few granules were formed with only GRB2 or SHC1. This increased abundance of granules coincided with enhanced activation of the Ras/MAPK signaling pathway. It was worth noting that the number of protein granules containing GRB2 and CCDC6-RET increased remarkably (Fig. 5 *E* and *F*). As is well known, SHC1 can be phosphorylated by autophosphorylated RET kinase via the binding of the CH1 domain or PTB domain to RET Y1062 and then acts as the docking site for GRB2 which was an adaptor protein linking cell surface kinase receptors to Ras/MAPK signaling effector (51). However, the precise interaction between GRB2 and RET remains unclear. Some studies have suggested that GRB2 could bind to the RET long isoform (RET51) via Y1096 with low affinity (52). Additionally, in 1998, *Alberti* reported that GRB2 could bind to the Y620 of RET/PTC2 (corresponding to Tyr1096 on proto-RET), suggesting that the binding of RET and GRB2 was not only mediated by SHC1 but also by other molecules (53).

In our research, we made an intriguing finding that GRB2 exhibited colocalization with CCDC6-RET, albeit with weak colocalization observed with RET-FL (Fig. 5*G*). Based on this observation, we hypothesized that GRB2 could bind to CCDC6-RET with stronger interaction. To validate this hypothesis, we performed CRY2-induction and coimmunoprecipitation experiments to investigate their direct interaction. Upon induction with blue light, CCDC6-RET formed granules, and GRB2 was also observed to colocalize within these granules (*SI Appendix*, Fig. S5 *A* and *B*). Likewise, the significant interaction between CCDC6-RET and GRB2 was detected by the reciprocal pull-down assays (Fig. 5 *H*–*K*). These results confirm a direct and stronger intermolecular interaction between CCDC6-RET and GRB2 than previously reported. Subsequently, we investigated the typical mutants in both RET and GRB2. Both the enzymatic-loss mutant in CCDC6-RET (K147M, Δ21-27) and the function-loss mutant in GRB2 (ΔSH2 domain) resulted in the disappearance of protein granules, while the impact of the reported interaction-associated point mutants (CCDC6-RET Y485F and GRB2 R86K) was limited (Fig. 5 *L*–*P* and *SI Appendix*, Fig. S5 *C*–*F*). These results suggest that functional RET capable of undergoing LLPS and GRB2 with the SH2 domain is essential for formation of membraneless protein granules.

### The CCDC6-RET Recruits GRB2 and SHC1 Synchronously to Form the Signal Niche.

**Fig. 6.**
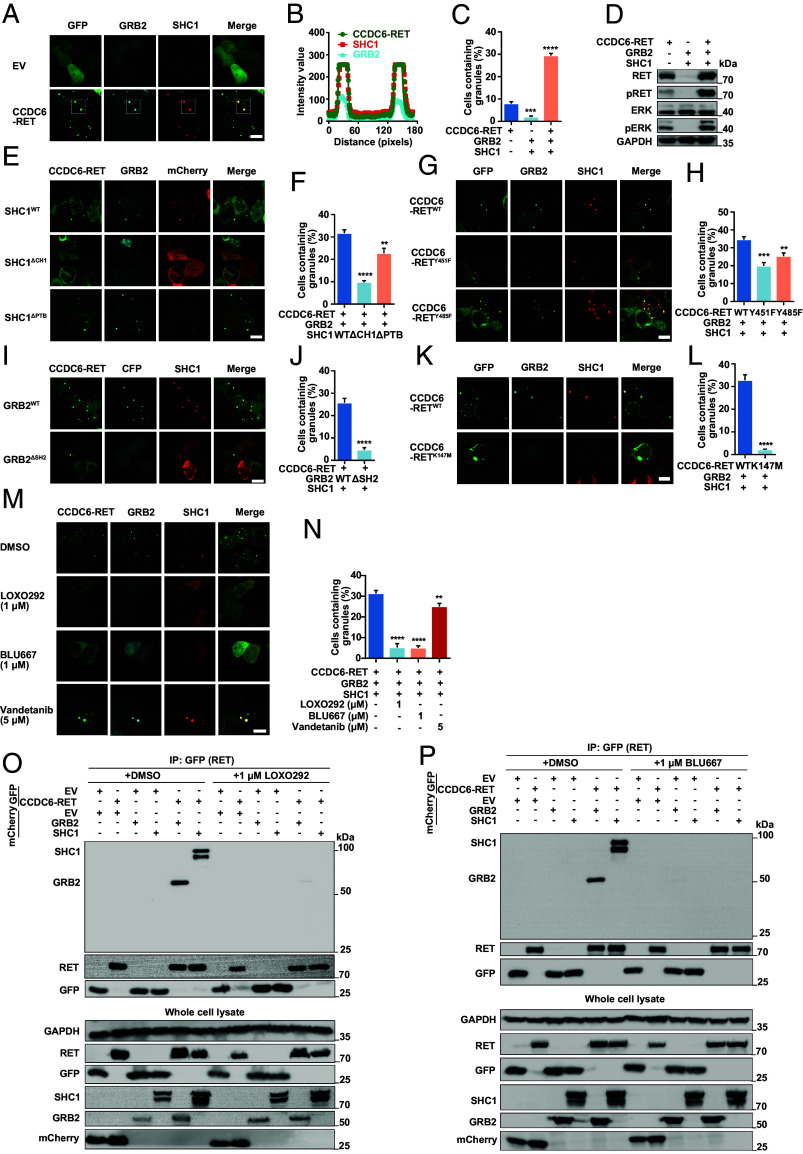
The Ras/MAPK signaling niche contains CCDC6-RET, GRB2, and SHC1 synchronously. (*A*–*D*) The cotransfection of CCDC6-RET-GRB2-SHC1 promotes the formation of protein granules and enhances the Ras/MAPK signal. Live-cell imaging of HEK293T cells with co-transfected CCDC6-RET-EGFP, GRB2-ECFP and SHC1-mCherry in *A*, the plot of the colocalization of CCDC6-RET, GRB2 and SHC1 in *B*, the analyzed foci is within the dashed square, the proportion of cells with protein granules in *C* and the Ras/MAPK signaling detected through western blotting in *D*, n ≥ 3. Error bars indicate SD from three regions in the imaged sample selected for counting, with each region containing more than 200 total cells. ****P < 0.0001 and ***P < 0.001 versus CCDC6-RET by Student’s *t* test. (Scale bar, 10 µm.) (*E* and *F*) The LLPS of cotransfection CCDC6-RET-GRB2 with SHC1 WT, ΔCH1, or ΔPTB mutants in HEK293T cells. Live-cell imaging in *E* and the proportion of cells with protein granules in *F*, n ≥ 3. Error bars indicate SD from three regions in the imaged sample selected for counting, with each region containing more than 200 total cells. ****P < 0.0001 and **P < 0.01 versus CCDC6-RET by Student’s *t* test. (Scale bar, 10 µm.) (*G* and *H*) The LLPS of cotransfection GRB2 and SHC1 with CCDC6-RET WT, Y451F, or Y485F mutants in HEK293T cells. Live-cell imaging in *G* and the proportion of cells with protein granules in *H*, n ≥ 3. Error bars indicate SD from three regions in the imaged sample selected for counting, with each region containing more than 200 total cells. ***P < 0.001 and **P < 0.01 versus CCDC6-RET by Student’s *t* test. (Scale bar, 10 µm.) (*I* and *J*) The LLPS of cotransfection CCDC6-RET and SHC1 with GRB2 WT or ΔSH2 mutant in HEK293T cells. Live-cell imaging in *I* and the proportion of cells with protein granules in *J*, n ≥ 3. Error bars indicate SD from three regions in the imaged sample selected for counting, with each region containing more than 200 total cells. ****P < 0.0001 versus CCDC6-RET by Student’s *t* test. (Scale bar, 10 µm.) (*K* and *L*) The LLPS of cotransfection GRB2 and SHC1 with CCDC6-RET WT, K147M mutant in HEK293T cells. Live-cell imaging in *K* and the proportion of cells with protein granules in *L*, n ≥ 3. Error bars indicate SD from three regions in the imaged sample selected for counting, with each region containing more than 200 total cells. ****P < 0.0001 versus CCDC6-RET by Student’s *t* test. (Scale bar, 10 µm.) (*M* and *N*) The LLPS of cotransfection CCDC6-RET, GRB2, and SHC1 in HEK293T cells treated with an equivalent volume of drug solvent (DMSO), LOXO292 (1 µM), BLU667 (1 µM), and vandetanib (5 µM). Live-cell imaging in *M* and the proportion of cells with protein granules in *N*, n ≥ 3. Error bars indicate SD from three regions in the imaged sample selected for counting, with each region containing more than 200 total cells. ****P < 0.0001 and **P < 0.01 versus solvent by Student’s *t* test. (Scale bar, 10 µm.) (*O* and *P*) Anti-GFP CO-IP of EGFP-tagged CCDC6-RET or EV transfected in HEK293T cells with coexpression mCherry-tagged GRB2, SHC1, or EV treated with LOXO292 (1 µM) in *O* and BLU667 (1 µM) in *P*, respectively, followed by western blotting. The control group was treated with an equivalent volume of drug solvent, DMSO.

Ably, the number of protein granules prominently increased in conjunction with the enhanced activation of Ras/MAPK signaling, following the simultaneous transfection of CCDC6-RET, GRB2, and SHC1 into cells (Fig. 6 *A*–*D* and *SI Appendix*, Fig. S6 *A*–*C*). Inspired by these results, we proposed that CCDC6-RET can recruit GRB2 and SHC1 synchronously to form the signaling niche through LLPS, thereby facilitating the transmission of the Ras/MAPK signal. To explore the functional roles of these three proteins within the signal niche, we generated a series of domain-specific mutants. SHC1 can bind to phosphorylated RET directly through the CH1 or PTB domain (54). We observed that the absence of the CH1 or PTB domain had negligible impact on the formation of protein granules containing CCDC6-RET and SHC1, presumably due to the formation of only a limited number of granules (*SI Appendix*, Fig. S6 *D* and *E*). However, the absence of the CH1 domain had a notable impact on the formation of the three-component signal niche, whereas the lack of the PTB domain did not exhibit a similar effect (Fig. 6 *E* and *F*). The finding suggests that the CH1 domain of SHC1 plays a more crucial role in this process compared to the PTB domain.

The point mutation in SHC1 Y239, the critical site for interacting with GRB2, brought minimal impact on both the quantity of granules and the transfection of the Ras/MAPK signaling pathway (*SI Appendix*, Fig. S6 *F*–*H*). Both GRB2 and SHC1 can interact with RET at the phosphorylated residues Y1096 and Y1062 through the SH2 domain in GRB2 and the CH1/PTB domain in SHC1 (55–57). We observed that the CCDC6-RET Y485F and Y451F mutants led to a reduction in the formation of ternary membraneless organelles. Additionally, the GRB2 mutant with the SH2 domain deficient almost completely eliminated the signal niche granules, resulting in the absence of Ras/MAPK signal transduction (Fig. 6 *G*–*J*). The interaction between GRB2 and tyrosine-phosphorylated target proteins is facilitated by its SH2 domain, which plays a crucial role in mediating protein–protein interactions of functional proteins. The SH2 domain serves as a critical mediator between receptor and kinase (58). As expected, our results showed that neither LLPS-related mutant (Δ21-27) nor the kinase activity-defection mutants (K147M) were able to form the signal niche in conjunction with GRB2 and SHC1 (Fig. 6 *K* and *L* and *SI Appendix*, Fig. S6*I*). Additionally, we observed a direct correlation between the LLPS ability of RET fusion proteins and their capacity to form signal niche (*SI Appendix*, Fig. S6 *J*–*L*). Overall, in the CCDC6-RET-GRB2-SHC1 signal niche, the CCDC6-RET phase separation and the intact SH2 domain in GRB2 were indispensable for maintaining the stability of membraneless signal niches and regulating the Ras/MAPK signaling pathway.

**Fig. 7.**
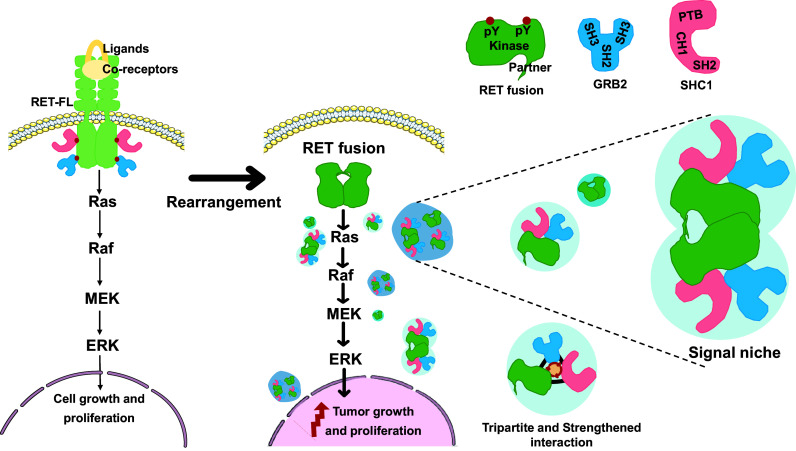
Model for CCDC6-RET-GRB2-SHC1 ternary LLPS signal niche.

Furthermore, we investigated whether the formation of LLPS signal niches exhibited a universal pattern in RET fusion and its interacting proteins, such as SHP2, FRS2, and SOS1, which were identified as components enriched in RET fusion protein granules (37, 59, 60). Notably, SHP2, SOS1, and SHC1 are all capable of binding to GRB2 and facilitating Ras/MAPK signaling transmission (37). The same colocalization results have also been observed in our research. However, except for SHC1, adding other proteins to the RET-fusion granules did not result in the formation of additional granules (*SI Appendix*, Fig. S6*M*). These results demonstrate that CCDC6-RET undergoes LLPS to recruit the adaptor protein GRB2 and the effector protein SHC1, resulting in the formation of signal niches characterized by ternary interactions (Fig. 7). Interestingly, the stability of membraneless signal niche is distinctly unique, playing an important role in the Ras/MAPK signaling pathway. Perturbation of either component within this triangle relationship results in a reduced number of signal niches, underscoring the indispensability of both the normal enzymatic activity of RET and the intact SH2 domain of GRB2 in this process.

In addition, we explored the potential of RET inhibitors to disrupt the CCDC6-RET-GRB2-SHC1 complex. We assessed the effects of clinically approved drugs, including the recently developed LOXO292 and BLU667, as well as the traditional multikinase inhibitor vandetanib (31, 61, 62). Our findings indicate that LOXO292 and BLU667 can effectively inhibit the Ras/Mapk signaling at concentrations as low as 1 µM, whereas vandetanib shows partial blocking of this signaling pathway at higher concentrations of 5 µM (*SI Appendix*, Fig. S7 *A*–*L*). Concurrently, our observations focused on whether these inhibitors could disrupt the specific CCDC6-RET-GRB2-SHC1 signal niche. Consistent with the western blot results (*SI Appendix*, Fig. S7 *D*, *H*, and *L*), BLU667 and LOXO292 significantly reduced the interaction between CCDC6-RET and GRB2 or SHC1, markedly diminishing the signal niche (Fig. 6 *M*–*P*). These three inhibitors, including LOXO292, BLU667, and vandetanib, are ATP-competitive inhibitors. They function by binding to the ATP binding site of the kinases, which subsequently inhibits kinase activity and downstream signaling pathways. Our study also provides evidence that interference with the kinase activity of CCDC6-RET can significantly influence the formation of CCDC6-RET-GRB2-SHC1 signal niche. Consequently, it seems that these typical RET inhibitors disrupt signal niches primarily by inhibiting the enzyme activity of CCDC6-RET, rather than other potential mechanisms, such as directly disrupting the protein–protein interaction.

## Discussion

RET is an oncogene responsible for encoding the oncoprotein, which activates various tumorigenic signaling pathways. Among these pathways, the Ras/MAPK signaling pathway stands out as the most frequently observed hallmark (33). The aberrant expression of RET, including somatic point mutations and cytogenetic rearrangements, results in the dysregulation of mitogenic signaling pathways (63). The underlying pathogenic mechanisms for the majority of somatic point mutants have been elucidated. For instance, the C634R mutation alters the intracellular dimerization process, while the V804L mutation interferes with prognosis. With the development of detection techniques, more and more RET fusion genes have been detected (64–66). The prevailing consensus suggests that these rearranged RET proteins exhibit ligand-independent constitutive kinase activity and function similarly to RET-FL. However, the efficacy of RET-targeting inhibitors appears to be diminished when targeting these rearranged variants (67). Recent studies have shown that fusion kinases, such as EML4-ALK, and CCDC6-RET, could form membraneless protein granules with interactional pathway-related proteins to organize oncogenic Ras/MAPK signaling (37).

In this article, we reported that CCDC6-RET fusion protein could undergo liquid–liquid phase separation. We provided the biochemical and biophysical basis of CCDC6 fragment for LLPS both in cells and in vitro (Fig. 1 and *SI Appendix*, Fig. S1). Importantly, we demonstrate that the LLPS capability of the CCDC6-101aa and the intact kinase domain are indispensable for facilitating LLPS of the functional RET fusion protein (Fig. 3 and *SI Appendix*, Fig. S3). Membrane-localized tyrosine kinase can recruit adaptor and effector proteins, such as GRB2, SHC1, and SHP2, among others, to activate downstream signals, while the cytoplasmic rearrangement proteins can form membraneless protein granules with RAS-activating complex comprising GRB2/GAB1/SOS1 (33, 37). On this basis, we found that the LLPS of functional CCDC6-RET is critical for the assembly of membraneless protein granules (Fig. 4 and *SI Appendix*, Fig. S4). Within the Ras/MAPK signaling pathway, GRB2 and SHC1 play important roles as adaptor and effector proteins, directly interacting with RET. In our live-cell imaging observation, we observed a higher number of protein granules formed between CCDC6-RET and GRB2 in comparison to SHC1. In contrast to the relatively weak binding affinity between RET and GRB2, GRB2 appears to exhibit a stronger affinity with CCDC6-RET. We proposed that GRB2 facilitates the LLPS of CCDC6-RET which in turn strengthens the interaction between them (Fig. 5 and *SI Appendix*, Fig. S5). Notably, our findings reveal that the CCDC6-RET recruits both GRB2 and SHC1 to form a specialized signaling niche, leading to an amplification of Ras/MAPK signaling, characterized by a significant increase in the number of granules. The pairwise interactions among CCDC6-RET, GRB2, and SHC1 are interconnected and mutually dependent. Each protein relies on its interaction with the other two proteins to form a stable and functional signal niche. Disrupting any of these interactions would likely destabilize the entire complex and impair the ability of the signal niche to transduce the Ras/MAPK signaling cascade (Figs. 6 and 7 and *SI Appendix*, Fig. S6). Furthermore, we speculate that the heightened transforming capacity observed in the recently reported RET-GRB2 fusion may be attributed to a stronger interaction between RET and GRB2, which arises from the direct linkage between the RET kinase domain and the GRB2 SH2 domain. The cooperative interaction between the RET fusion and GRB2 plays a dominant role within this signaling niche, surpassing other interactions.

The distinctive CCDC6-RET-GRB2-SHC1 granules observed in our work resemble a cytoplasmic signal niche to enrich signaling effectors, which then activate the signaling cascade without the need of upstream ligand stimulation. In comparison to the naturally membrane-localized RET, the flexible subcellular localization of CCDC6-RET and the presence of disordered regions within the fusion partner provide an opportunity for the recruitment of multiple interacting proteins, forming an unconstrained membraneless niche. The formation of this signal niche is contingent upon the kinase activity of CCDC6-RET and its interaction with GRB2 and SHC1. Disruption of the kinase activity of CCDC6-RET, either through mutagenesis in the fusion partner or the kinase domain, impedes the formation of signal niche and blocks the signaling pathway. In our further exploration of the role of kinase activity in this process, we utilized clinical inhibitors such as LOXO292, BLU667, and vandetanib. These inhibitors function by binding to the ATP-binding site of the kinase, thereby inhibiting its activity (31, 68). We found that these inhibitors disrupt the interaction between CCDC6-RET and either GRB2 or SHC1, which affects the formation of signal niche and ultimately leads to the blockade or attenuation of the signaling pathway transduction. Interestingly, when we selectively disrupted the interaction between CCDC6-RET and either GRB2 or SHC1 by deleting the crucial protein–protein binding region without altering the enzymatic activity of CCDC6-RET, we observed a disruption in the formation of the signal niche and an inhibition of the Ras/MAPK signaling pathway. This suggests that to inhibit the Ras/MAPK signaling pathway, we can either disrupt the protein–protein interaction within the CCDC6-RET-GRB2-SHC1 complex without inhibiting the kinase activity of CCDC6-RET, or directly inhibit the kinase activity of RET. Currently, most existing RET inhibitors primarily function by targeting kinase activity. The design of these kinase inhibitors is largely informed by the structural features of kinases, with inhibitors commonly targeting specific sites or pockets within the kinase domain. However, the design of kinase inhibitors encounters substantial challenges in precisely targeting various kinases due to the similarity of active sites (69). Our results suggest a promising approach: selectively disrupting the interaction between CCDC6-RET and either GRB2 or SHC1 to hinder signal niche formation. This disruption subsequently leads to the inhibition of signal transduction, potentially addressing the issue of poor specificity. Consequently, the identification of specific compounds that can disrupt the ternary complex and selectively accumulate within the CCDC6-RET condensate may provide a unique opportunity for targeted therapies against RET fusion–driven cancers. Current drug research in the field of aberrant phase separation is rapidly advancing. Recent studies indicate the feasibility of identifying compounds that can modulate abnormal condensates through high-throughput drug screening methods (70). Alternatively, the use of proteolysis-targeting chimeras (PROTACs) to induce degradation of phase separation proteins is also being explored (71). The concept of CCDC6-RET-GRB2-SHC1 holds the potential to accelerate the development of CCDC6-RET targeted therapy by leveraging targeted phase separation mechanisms.

However, several aspects remain unanswered and merit further investigation within the scope of our study. Specifically, the composition of the CCDC6-RET-GRB2-SHC1 signal niche warrants deeper exploration, as our research has identified the presence of CCDC6-RET, GRB2, and SHC1 within the niche. Still, it is conceivable that additional components contribute to its complexity. Additionally, the mechanism underlying the interaction between the signal niche and downstream targets in the signaling pathway remains elusive. One possibility is that the target proteins in the downstream pathway are recruited into the signal niche in a manner similar to the membrane-locate RET, which recruits target proteins to lipid membrane. Alternatively, the signal niche may act as a dynamic entity, relocating in proximity to the target proteins. Advancing our understanding of the dynamic nature and constituent elements of the signal niche, as well as its communication with downstream targets, will provide valuable insights into the molecular mechanisms governing the aberrant Ras/MAPK signaling cascade driven by the CCDC6-RET fusion. Furthermore, we found that a significant proportion of RET fusion proteins, including KIF5B-RET and NCOA4-RET, possess the coiled-coil domain within their fusion partners (72–74). This observation raises the intriguing possibility that RET fusion proteins may recruit signaling proteins via LLPS, thereby contributing to the promotion of cancers. Exploring this phenomenon represents a valuable direction for further investigation and will provide insights into the understanding of tumorigenesis driven by RET fusion proteins.

## Materials and Methods

Detailed materials and methods are provided in *SI Appendix*. Cells exhibiting stable expression of fusion proteins, such as TPC-1 and 293T stable cell lines, were identified using western blot analysis. Stable cell lines expressing CCDC6-RET were generated through lentivirus infection. The transient expression process was conducted using Lipo8000™ or Lipo3000. In vitro, recombinant proteins were produced in *Escherichia coli* BL21 (DE3) cells. The analysis of phase separation involved turbidimetry, DIC, and confocal microscope observation. Statistical assessments, performed with Student’s *t* test or one-way ANOVA, compared groups using GraphPad Prism software. Colocalization analyses were performed using the “Plot Profile” in Image J software. All data are included in the manuscript and *SI Appendix*.

## Supplementary Material

Appendix 01 (PDF)

Movie S1. **Time-lapse imaging of EGFP-tagged CCDC6 and CCDC6-Δ101aa droplet fusion, related to Fig. 1**. (A) A video presentation of CCDC6 protein droplets fusion in cells.

Movie S2. **Time-lapse imaging of EGFP-tagged CCDC6 and CCDC6-Δ101aa droplet fusion, related to Fig. 1**. (B) A video presentation of CCDC6-Δ101aa protein droplets fusion in cells.

## Data Availability

All study data are included in the article and/or supporting information.
