# Supplementary material for: CCDC6-RET fusion protein regulates Ras/MAPK signaling through the fusion- GRB2-SHC1 signal niche
Source: Proc Natl Acad Sci U S A. 2024 May 28;121(23):e2322359121. doi: 10.1073/pnas.2322359121 (PMC11161787; doi:10.1073/pnas.2322359121)
Supplement: Supplementary file 1 — Appendix 01 (PDF) [file pnas.2322359121.sapp.pdf]

PNAS

Supporting Information for

CCDC6-RET fusion protein regulates Ras/MAPK signaling through the fusion-GRB2-SHC1 signal niche

Ting Qiu[a,b,c,d,1,*], Yichao Kong[a,b,c,d,1], Guifeng Wei[a,b,c,d,1], Kai Sun[a,b,c,d], Ruijie Wang[a,b,c,d], Yang Wang[a,b,c,d], Yiji Chen[a,b,c,d], Wenxin Wang[a,b,c,d], Yun Zhang[a,b,c,d,e], Caihong Jiang[a,b,c,d], Peiguo Yang[e], Tian Xie[a,b,c,d,*], Xiabin Chen[a,b,c,d,*]

*Ting Qiu, Tian Xie, Xiabin Chen.
Email: tingqiu@hznu.edu.cn, or xbs@hznu.edu.cn, or xch226@hznu.edu.cn

This PDF file includes:

Materials and Methods
Figures S1 to S6
Legends for Movies S1
SI References

Other supporting materials for this manuscript include the following:

Movies S1

Materials and Methods

Cell lines

HEK293T, Hela, BEAS-2B, H1299 and TPC-1 cells were used for this study. HEK293T and Hela cells were generous gift from Yusheng Cong lab (Hangzhou Normal University, China). BEAS-2B cells were obtained from FENGHBIO (CL0044, China), which were authenticated by STR profiling by the supplier. Cells above were cultured in DMEM (Vistech) supplemented with 10% FBS (Vistech) and 1% PS (Gibco). H1299 cells were generous gift from Xinbing Sui lab (Hangzhou Normal University, China), and cultured in RPMI-1640 medium (VISTECH) supplemented with 10% FBS and 1% PS. And TPC-1 cells were purchased from Shanghai Chuan Qiu Biotechnology (H060, China) and also authenticated by STR profiling by the supplier. TPC-1 cells were cultured in Ham's F-12K (Kaighn's) (Gibco) with 15% FBS and 1% Antibiotic-Antimycotic (Gibco). We have detected the CCDC6-RET in TPC-1 cells using PCR and WB (*SI Appendix*, Fig S2*A* and S2*B*). All cell lines were maintained at 37 °C with 95% air and 5% CO_2. *Escherichia. coli* DH5α (Tsingke Biotechnology) and BL21(DE3) (Tsingke Biotechnology) competent cells were cultured in LB media with the appropriate antibiotic at 37°C in a shaker incubator (200 rpm).

Antibodies and reagents

Antibodies against RET (#3223), phospho-RET (Tyr905) (#3221), MEK1/2 (#9122), Phospho-MEK1/2 (Ser217/221) (41G9) (#9154), p44/42 MAPK (ERK1/2) (137F5) (#4695), Phospho-p44/42 MAPK (ERK1/2) (Thr202/Tyr204) (#9101), DYKDDDDK Tag (#8146), β-Tubulin (D3U1W) (#86298), mCherry (E5D8F) (#43590) and GAPDH (14C10) (#2118) were purchased from Cell Signaling Technology (USA). Anti-PLCγ1 (#sc-7290), anti- p-PLCγ1 (#sc-136186), anti- SHC (#sc-967), anti-GRB2 (#sc-8034), anti-CCDC6 (Q-23) (#sc-100309) and anti-GFP (#sc-9996) were purchased from Santa Cruz Biotechnologies (USA). β-actin (#BK7018), HRP-Goat Anti-rabbit IgG (H+L) (#BK-R050) and HRP-Goat Anti-mouse IgG (H+L) (#BK-M050) were purchased from BIOKER (Hangzhou, China). All antibodies were used at a dilution of 1:1000 except for β-Tubulin, β-actin and GAPDH, which were used at dilutions of 1:3000. Hoechst (#BK-R050) was obtained from Aladdin (Shanghai, China). BLU667 (#HY-112301), LOXO292 (#HY-114370) and vandetanib (#HY-10260) were purchased from MedChemExpress (USA).

Cell transient transfection

HEK293T, Hela, BEAS-2B and H1299 cells transfected by using Lipo8000™ Transfection Reagent (Beyotime) following the manufacturer's protocol. Transfection of TPC-1 cells were performed using Lipofectamine 3000 (Thermo Fisher) for transient transfections following the manufacturer's instructions.

Cell lines generation

HEK293T cells were utilized to establish stable cell lines expressing CCDC6-RET-EGFP or EGFP through lentiviral transduction. The pLVX-CCDC6-RET-EGFP or pLVX-IRES-Puro plasmids (Generous gift from Jianxiang Chen lab, Hangzhou Normal University, China) were used for transfection. HEK293T cells, were seeded at a density of $5×10^6$ cells per 10 cm dish and transfected with pLVX-CCDC6-RET-EGFP or pLVX-IRES-Puro, pMDLg/pRRE, pMD2.G and pRSV-Rev in a ratio of 10:10:5:2 to get the virus. HEK293T cells were infected with the appropriate concentration of virus along with 5 µg/mL polybrene for 4-6 hours and selected with optimal puromycin dihydrochloride after 48h. Before using, the stable cell line was validated by western blot and microscopic observation. To generate knock-in stable cell lines, TPC-1 cells were grown in a 6-well plate and transfected with CRISPR/Cas9 and donor plasmids using Lipofectamine 3000. Two days after transfection, cells were selected in the presence of 1 mg/mL puromycin for three days. Before using, the stable cell line was validated by PCR and microscopic observation.

Plasmid Construction

DNA fragments encoding human CCDC6, RET, GRB2, SHC1 (p52SHC1), RET, KIF5B and NCOA4 were PCR-amplified from HEK293T, Hela cDNA libraries. The plasmids encoding CCDC6 and CCDC6-RET used in *Escherichia. coli* was synthesized by Generay Biotechnology (Shanghai, China).

To generate various constructs, the DNA fragments were cloned into the pET-32a vector to generate constructs. Meanwhile, they were also cloned into the pEGFP-N1 vector to generate constructs with a GFP tag using ClonExpress® II One Step Cloning Kit (Vazym). Deletion mutants of CCDC6-101aa were created using the KLD Enzyme Mix (NEB). CCDC6 sequence cDNA from bases 1-1422, mutants or deletions bases based on this sequence numbering. CCDC6-101aa: bases 1-303; CCDC6-150aa: bases 1-450; CCDC6-293aa: bases 1-879; CCDC6-Δ101aa: bases 304-1422; CCDC6-101aa$^{\Delta 21\text{-}27}$: deletion of bases 61-81; CCDC6-101aa$^{\Delta 49\text{-}51}$: deletion of bases 145-153; CCDC6-101aa$^{\Delta 70\text{-}77}$: deletion of bases 208-231; CCDC6-101aa$^{\Delta ployG}$: deletion of bases 94-132.

DNA fragments encoding CCDC6-RET or its mutants were inserted into the pEGFP-N1 vector. To generate stable cell lines, CCDC6-RET-coding DNA was ligated into the pLVX-GFP-IRES-Puro plasmid. To generate CRY2-oligo-tagged plasmids, the coding sequences of selected proteins was cloned into CRY2oligo-mCherry vector (Generous gift from Peiguo Yang lab, Westlake University, China). CCDC6-RET was constructed by fusing bases 1-303 of CCDC6 with the RET kinase domain (RET-KD) (bases 2137-3342 of RET). Mutations or deletions were made based on this sequence numbering. CCDC6-RET K147M: RET K758M mutant; CCDC6-RET Y294F/ CCDC6-RET Y370F/ CCDC6-RET Y404F/ CCDC6-RET Y451F/ CCDC6-RET Y485F as followed RET Y905F, Y981F, Y1015F, Y1062F and Y1096F mutants; CCDC6-150aa-RET: bases 1-450 of CCDC6 fused to RET-KD; CCDC6-293aa-RET: bases 1-879 of fused to RET-KD; KIF5B-RET/NCOA4-RET were constructed using bases 1-1725 of KIF5B or 1-762 of NCOA4 fused to the RET-KD.

The cDNA sequence for GRB2 or SHC1 (p52SHC1) were cloned into the pEGFP-N1 vector. At the same time, C-terminal tags were replaced with EYFP or mCherry according to the experimental needs. The mutants or deletions bases based on sequence numbering of GRB2 (bases 1-651) or SHC1 (bases 331-1749). GRB2R86K: GRB2 R86K mutant; GRB2ΔSH2: deletion of bases 178-456. SHC1Y239F: SHC1 Y239F mutant; SHC1ΔPTB: deletion of bases 136-687; SHC1ΔCH1: deletion of bases 688-1134.

To tag endogenous CCDC6-RET in TPC-1 cells using the CRISPR/Cas9 technology, the pCE3 Blunt Vector and px459 VQR (101715, Addgene, USA) were employed according to the protocol of Feng Zhang(1, 2). sgRNA sequences are as follows: gRNA1: 5'-GCAAAATTAATGGACACGTT.

Cell lines identification

To identify the expression of CCDC6-RET, CCDC6 and RET-FL gene in TPC-1 and 293T cell lines, PCR was conducted using the following primers: 5'-GCATTGTCATCTCGCCGTTCC-3' and 5'-CTGTGAGATCTGCCAGGCAAATGAG-3' for CCDC6-RET; 5'-GCATTGTCATCTCGCCGTTCC-3' and 5'-GCTTCTTCAGCCCCGTTCCACT-3' for CCDC6; 5'-AGACATCAACATTTGCCCTCA-3' and 5'-GCCGTTGCCTTGACCACTTTTC-3' for RET. The PCR utilized cDNA extracted from each cell line as a template. To identify TPC-1 knockin-mClover cell lines, genomic DNA from knock-in and wild-type cell lines. These DNA samples were then used as templates for PCR analysis targeting the CCDC6-RET-mClover region, employing the specified following primers: 5'- CAAGCTGAGACGGTTCCTTTTGCAT-3' and 5' TACTTGTACAGCTCGTCCATGCC-3'.

Protein Purification

For the phase-separation assay in vitro, CCDC6-FL, CCDC6-Δ101aa, CCDC6-101aa, CCDC6-150aa, CCDC6-293aa and various CCDC6-101aa mutants were expressed in *Escherichia. coli* BL21 (DE3) cells. The cells were induced with 1mM IPTG and incubated overnight at 17 °C. After

pelleting the cells, they were resuspended in 50 mM Tris-HCl (pH 8.0), 500 mM NaCl, 5% Glycerol buffer and lysed using a French press (Thermo Fisher Scientific). The protein was purified using Ni-affinity chromatography. The elution fractions were analyzed for quantity and purity using SDS-PAGE, and the desired fractions were concentrated using an Amicon Ultra-30 K centrifuge (Millipore). Finally, the purified protein was stored at -80 °C until further use.

Liquid-Liquid Phase Separation in vitro

Before the phase separation assay in vitro, the protein concentration was determined using the Enhanced BCA Protein Assay Kit. The LLPS of proteins were examined by addition of PEG 8000 (Sigma-Aldrich) as gradient change. The samples were transferred to a chamber using a double-sided tape between the slide and the cover slip (Invitrogen). The DIC microscope (Olympus IX73) was used to observe with a 40x objective. Comparing phase separation behaviors between CCDC6, CCDC6-101aa and CCDC6-Δ101aa, images were taken by Olympus FV3000 microscope with Z-stacking at a concentration of 8 μM with 5% PEG 8000. 15 images were taken with 4 μm stepsize in order to create a three-dimensional image. The following analyses were done in Cellsens software.

Live-cell microscopy

For live-cell confocal microscopy, cells were seeded in 20 mm diameter imaging dishes. After transfection 48h, cells were imaged using Olympus FV3000 microscope (excitation DAPI/ECFP: 405 nm, excitation EGFP/EYFP: 488 nm, excitation mCherry: 587 nm) with 60x objective, maintained at 37 °C with 5% CO_2 using a live-cell station (STXG-IX3WX-SET, Tokai Hit). To obtain > 200 cells per condition, images were collected using 3×3 or 4×4 maps on FV31S-SW software.

For the light induction assay, cells were cultured under dark conditions for 48 hours after transgene expression. For the activation of condensates of CRY2oligo-mCherry, images were captured at 5 seconds intervals lasting approximately 10-20 minutes, which illuminated the cells with 405 nm and 488 nm lasers with 15%-20% power. Images were subsequently analyzed using ImageJ software.

Fluorescence Recovery After Photobleaching (FRAP)

For photobleaching experiments, cells were transfected with the relevant plasmids 48 hours prior to imaging. In order to FRAP of droplets, a 488 nm laser at 20~50% power for 1second were performed to photobleach regions of interest (ROIs) corresponding to individual granules in the sample using 60×. Time-lapse images remained over 0.5~10 minutes after bleaching with 5-10 seconds intervals. Further intensity analyses of bleached condensates were done in Origin 9 Prism and Cellsens software.

Turbidity Assay

In assess the turbidity of purified recombinant CCDC6, CCDC6-101aa and CCDC6-Δ101aa proteins, turbidity measurements were conducted using a Spark plate reader (Tecan) in a 384-well plate. Turbidity was measured at a wavelength of 350 nm. Protein samples were prepared in a final buffer containing 50 mM Tris-HCl (pH 8.0), 500 mM NaCl, 5% Glycerol buffer and 5% PEG 8000.

Western Blotting

Cells were seeded in 6/12-well plates and then transfected 48 hours for expression. Cells were lysed by RIPA buffer (Beyotime), added 1×phosphatase inhibitor cocktail A (Beyotime) and 1×PMSF. 5×SDS loading buffer was added into the lysates, then boiled at 95 °C for 10 min. Samples were separated in 4-12% SDS-PAGE gels and transferred the protein from the gel to the PVDF membrane using mini-trans blot device (Bio-rad). The membranes were blocked with 5% no-fat milk in TBST at room temperature, and then incubated the membrane with appropriate dilutions of primary antibody in Primary Antibody Dilution Buffer at 4 °C overnight. Signal was

detected using Enhanced Chemiluminescent (NCM Biotech) and chemiluminescence on eBlot Tough Image. For the light induction assay, cells were seeded in 96-well plates, transfected 24 hours for expression, illuminated by 405 and 488 nm lasers with 15%-20% power for 5 min/cycle remaining 3 cycles and followed by western blotting.

Co-immunoprecipitation (Co-IP)

HEK293T cells were transfected with either mCherry-tagged CCDC6-RET or EYFP-tagged versions of GRB2 and SHC1 in 6 cm dishes. After 48 hours of transfection, cells were lysed and scraped form the dishes, and then the lysate mixtures were centrifuged at 12000 rpm for 10 minutes at 4 °C. The supernatant was mixed with 15 µL mCherry trap beads (KT Life technology) at 4 °C overnight by 3D rotary mixer. The immunocomplexes were washed 3 times with IP buffer the following day and boiled mixing with 100 µL 1×SDS loading buffer at 95 °C for 10 minutes. The supernatant was analyzed by western blot.

Treatments of Cultured Cells with Drugs

All the compounds were stored and prepared according to the manufacturer's recommendation. BLU667, LOXO292 and vandetanib were added into the culture medium either 3 hours (for live cell imaging and CO-IP) or 24 hours (for western blotting) following cell transfection. Appropriate drug concentrations were applied to each dish(3-5). Live cell image, western blotting or Co-IP were performed 24 hours after drug treatment.

Statistical Analysis

For all the bar graphs, date represented mean ± SD or mean ± SEM, determined with by Student's t test or one-way ANOVA with between comparator groups using GraphPad Prism software. P values > 0.05 were considered no significant (ns). *, **, *** and **** represent $p <$ 0.05, 0.01, 0.001 and 0.0001, respectively. All experiments were independently repeated three times or more. The co-localization analyses were using 'Plot Profile' of Image J software.

Supplemental Figures

Fig. S1.

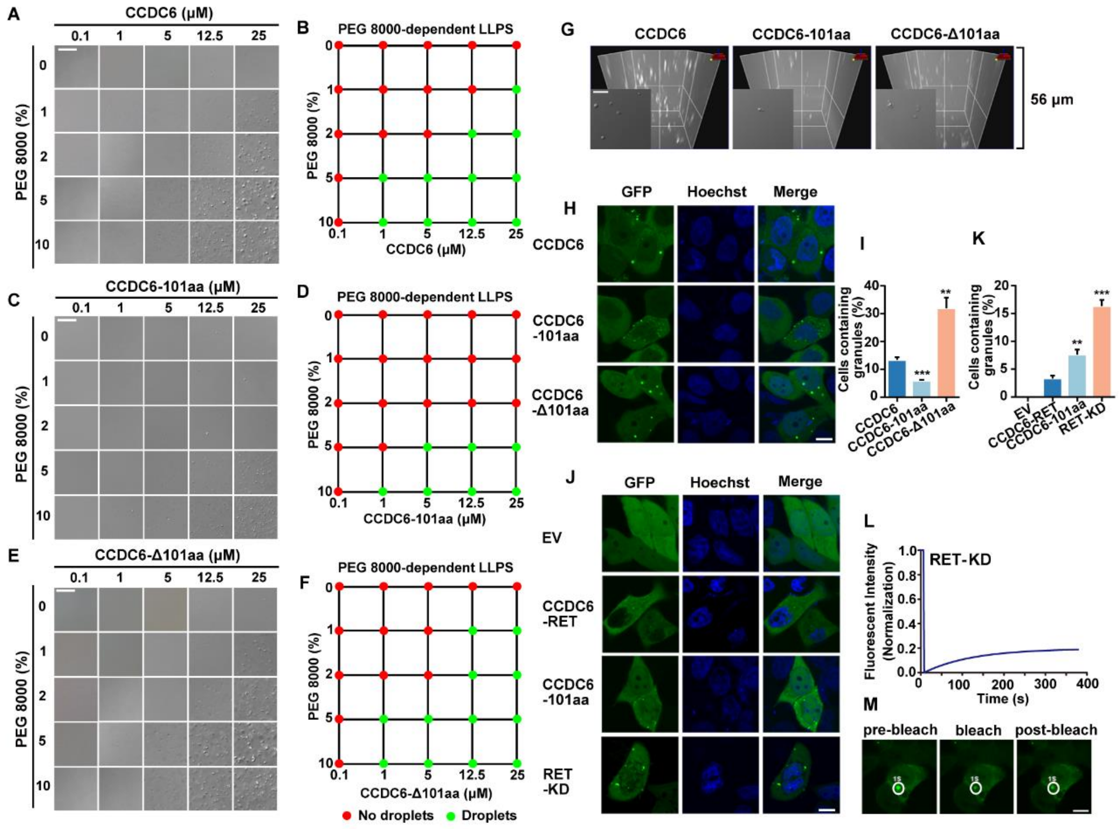

Fig. S1. The formation of different protein condensates in vitro and in cells, related to Fig. 1. **(A and B)** LLPS of purified recombinant CCDC6 protein was evaluated by the concentrations of protein and PEG 8000 in (A) and the summary result in (B). Scale bar, 20 μm. **(C and D)** LLPS of purified recombinant CCDC6-101aa protein was evaluated by the concentrations of protein and PEG 8000 in (C) and the summary result in (D). Scale bar, 20 μm. **(E and F)** LLPS of purified recombinant CCDC6-Δ101aa protein was evaluated by the concentrations of protein and PEG 8000 in (E) and the summary result in (F). Scale bar, 20 μm. **(G)** The images shown phase separation behaviors between CCDC6, CCDC6-101aa and CCDC6-Δ101aa were taken by confocal with Z-stacking at a concentration of 8 μM with 5% PEG 8000. The images were integrated into a 3D map. Z = 56 μm. Scale bar, 5 μm. **(H)** Live-cell imaging of EGFP-tagged CCDC6, CCDC6-101aa and CCDC6-Δ101aa expressed in Hela cells, respectively, n ≥ 3. Scale bar, 10 μm. **(I)** Proportion of cells with protein condensates upon transfection of CCDC6, CCDC6-101aa and CCDC6-Δ101aa in Hela cells. Error bars indicate SD from 3 randomly sampled regions with > 200 total cells per region. ***$p < 0.001$ and **$p < 0.01$ versus CCDC6 by Student's t test. **(J)** Live-cell imaging of EV and EGFP-tagged CCDC6-101aa, CCDC6-RET and RET-KD expressed in Hela cells, respectively, n ≥ 3. Scale bar, 10 μm. **(K)** Proportion of cells with protein condensates upon transfection of EV and EGFP-tagged CCDC6-101aa, CCDC6-RET and RET-KD in Hela cells. Error bars indicate SD from 3 randomly sampled regions with > 200 total cells per region. ***$p < 0.001$ and **$p < 0.01$versus CCDC6 by Student's t test. **(L and M)** FRAP of RET-KD-EGFP transfected in HEK293T cells. The FRAP curves are shown in (L) and corresponding droplet at bleached area in (M). Scale bar, 10 μm.

Fig. S2.

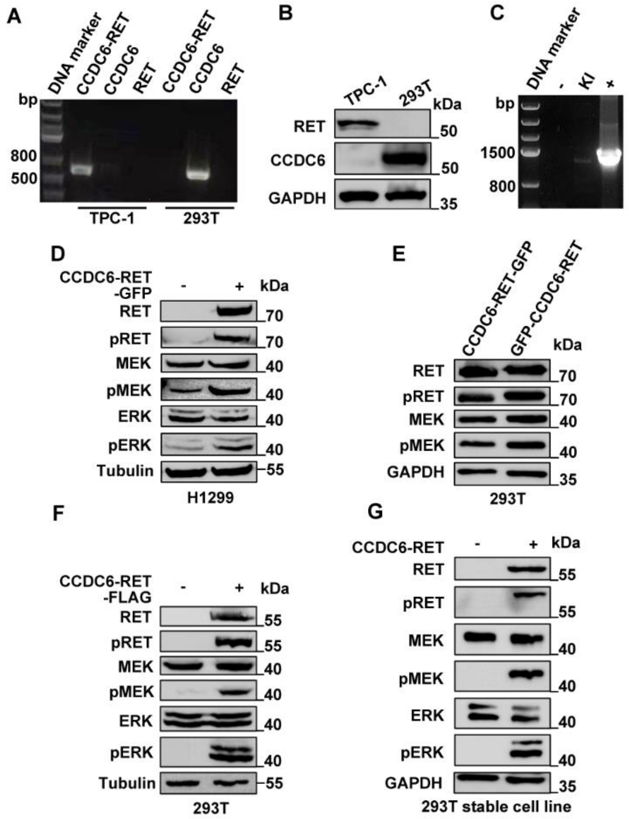

Fig. S2. Exogenous expression of CCDC6-RET drives the Ras/MAPK as the same with endogenous proteins, related to Fig. 2. **(A)** PCR analysis of CCDC6-RET, CCDC6 and RET-FL gene from TPC-1 and 293T cell lines. **(B)** Western blotting upon TPC-1 and 293T cell lines. **(C)** PCR analysis of mClover in endogenous mClover-tagging of CCDC6-RET of TPC-1 cell lines. -: TPC-1 cell lines; KI: mClover-tagging of CCDC6-RET of TPC-1 cell lines; +: plasmid T-Vector-HDR-CCDC6-RET-mClover. **(D)** Western blotting upon expression of EV or CCDC6-RET-EGFP in H1299 cells. **(E)** Western blotting upon expression of CCDC6-RET-EGFP or EGFP-CCDC6-RET in HEK293T cells. **(F)** Western blotting upon expression of EV or CCDC6-RET-Flag in HEK293T cells. **(G)** Western blotting upon HEK293T stable cell lines of EV or CCDC6-RET-EGFP.

Fig. S3

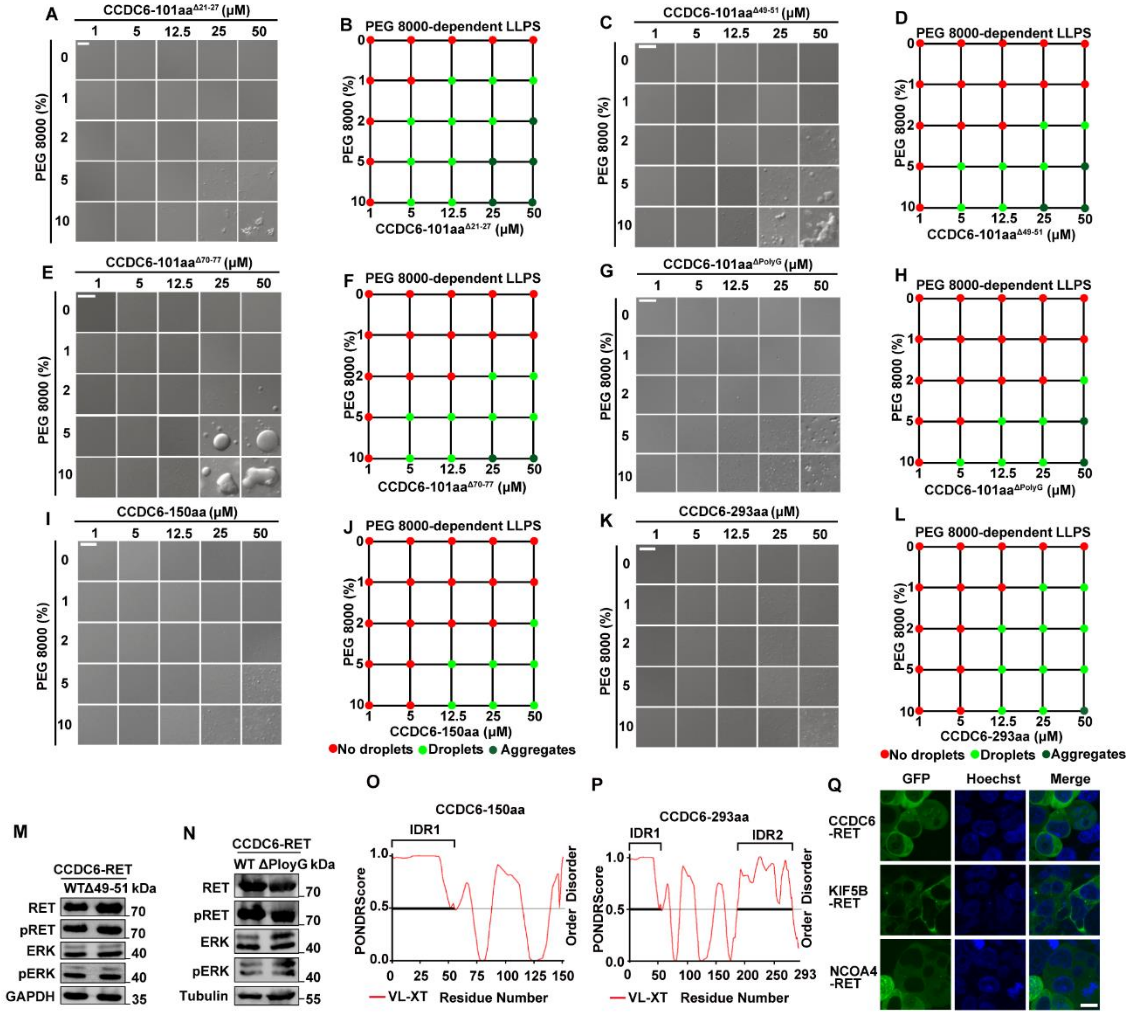

Fig. S3. The change of IDR directly affects the formation of protein condensates, related to Fig. 3. **(A, C, E and G)** LLPS of purified recombinant protein, including CCDC6-101aa$^{\Delta 21\text{-}27}$ in (A), CCDC6-101aa$^{\Delta 49\text{-}51}$ in (C), CCDC6-101aa$^{\Delta 70\text{-}77}$ in (E) and CCDC6-101aa$^{\Delta PloyG}$ in (G), was evaluated by the concentrations of protein and PEG 8000. Scale bar, 20 μm. **(B, D, F and H)** Phase diagram of CCDC6-101aa$^{\Delta 21\text{-}27}$ in (B), CCDC6-101aa$^{\Delta 49\text{-}51}$ in (D), CCDC6-101aa$^{\Delta 70\text{-}77}$ in (F) and CCDC6-101aa$^{\Delta PloyG}$ in (H) with increasing concentration of protein and PEG 8000. **(I and K)** LLPS of purified recombinant protein, including CCDC6-150aa in (I) and CCDC6-293aa in (K), was evaluated by the concentrations of protein and PEG 8000. Scale bar, 20 μm. **(J and L)** Phase diagram of CCDC6-150aa in (J) and CCDC6-293aa in (L) with increasing concentration of protein and PEG 8000. **(M)** Western blotting upon expression of EGFP-tagged CCDC6-RET or CCDC6-RET$^{\Delta 49\text{-}51}$ in HEK293T cells. **(N)** Western blotting upon expression of EGFP-tagged CCDC6-RET or CCDC6-RET$^{\Delta PloyG}$ in HEK293T cells. **(O and P)** The analysis of CCDC6-150aa in (O) and CCDC6-293aa in (P) with PONDR. **(Q)** Live-cell imaging of CCDC6-RET, KIF5B-RET and NCOA4-RET expressed in HEK293T cells, respectively, n $\geqslant$3. Scale bar, 10 μm.

Fig. S4

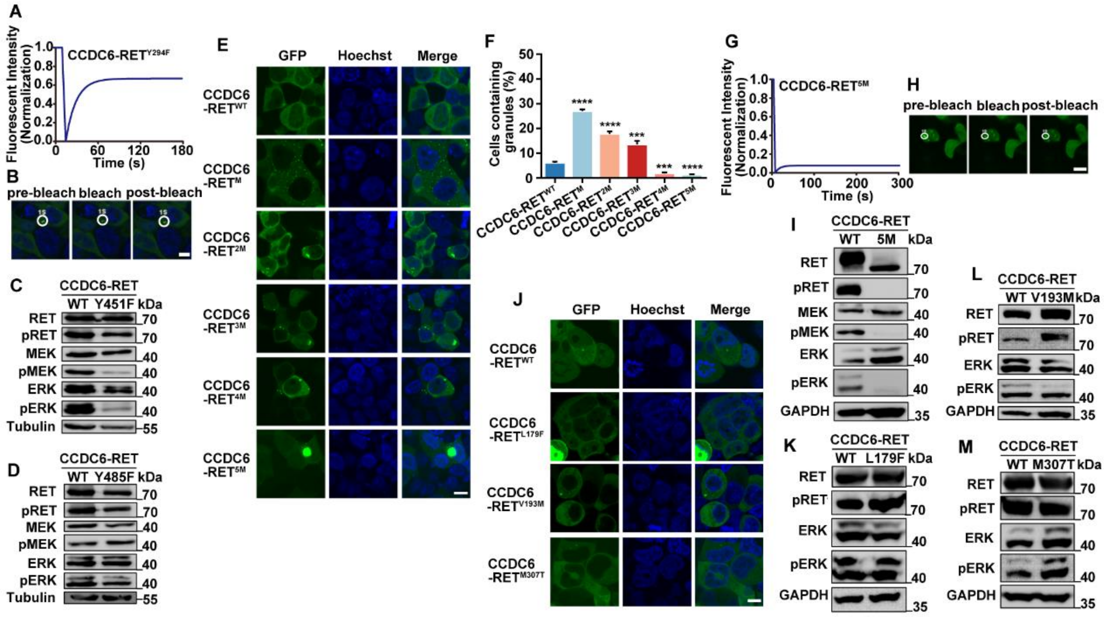

Fig. S4. Several mutants of RET in cancers have the different influences in the LLPS of fusion protein, related to Fig. 4. **(A and B)** FRAP of CCDC6-RET^{Y294F}-EGFP transfected in HEK293T cells. The FRAP curves in (A) and corresponding droplet at bleached area in (B). Scale bar, 5 µm. **(C)** Western blotting upon expression of CCDC6-RET-EGFP or CCDC6-RET^{Y451F}-EGFP mutant in HEK293T cells. **(D)** Western blotting upon expression of CCDC6-RET-EGFP or CCDC6-RET ^{Y485F} -EGFP mutant in HEK293T cells. **(E)** Live-cell imaging of CCDC6-RET-EGFP or its related multiple phosphorylation site mutations consisting of M (Y294F), 2M(Y294F/Y451F), 3M (Y294F/Y404F/Y451F), 4M (Y294F/Y370F/Y404F/Y451F) and 5M (Y294F/Y370F/Y404F/Y451F/Y485F) expressed in HEK293T, n ≥ 3. Scale bar, 10 µm. **(F)** Proportion of cells with protein condensates upon transfection of CCDC6-RET-EGFP or its related multiple phosphorylation site mutations consisting of M (Y294F), 2M (Y294F/Y451F), 3M (Y294F/Y404F/Y451F), 4M (Y294F/Y370F/Y404F/Y451F) and 5M (Y294F/Y370F/Y404F/Y451F/Y485F) in HEK293T cells. Error bars indicate SD from 3 randomly sampled regions with > 200 total cells per region. ****$p < 0.0001$ and ***$p < 0.001$ versus CCDC6-RET by Student's t test. **(G and H)** FRAP of CCDC6-RET^{5M}-EGFP transfected in HEK293T cells. The FRAP curves in (G) and corresponding droplet at bleached area in (H). Scale bar, 10 µm. **(I)** Western blotting upon expression of CCDC6-RET-EGFP or CCDC6-RET^{5M}-EGFP in HEK293T cells. **(J)** Live-cell imaging of CCDC6-RET-EGFP or its related site mutations consisting of L179F, V193M and M307T (RET L790F, V804M and M918T mutations) expressed in HEK293T, n ≥ 3. Scale bar, 10 µm. **(K)** Western blotting upon expression of CCDC6-RET-EGFP or CCDC6-RET^{L179F} -EGFP mutant in HEK293T cells. **(L)** Western blotting upon expression of CCDC6-RET-EGFP or CCDC6-RET^{V193M} -EGFP mutant in HEK293T cells. **(M)** Western blotting upon expression of CCDC6-RET-EGFP or CCDC6-RET ^{M307T}-EGFP mutant in HEK293T cells.

Fig S5.

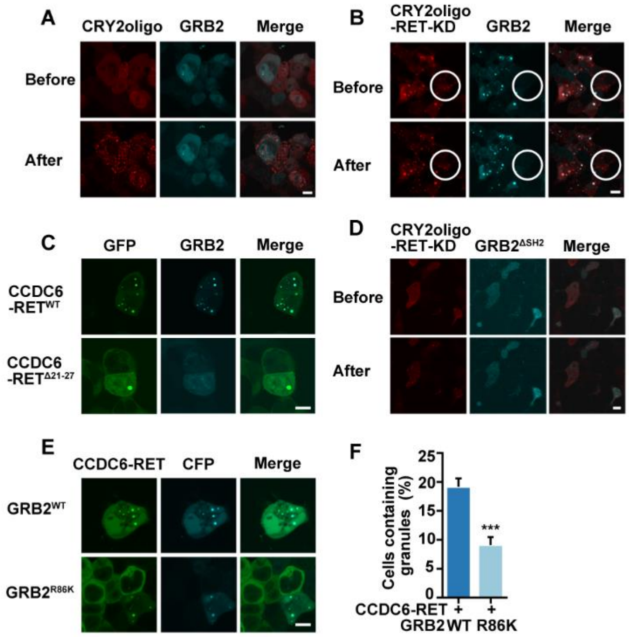

Fig. S5. Both CCDC6-RET and GRB2 are essential for the formation of signal granules, related to Fig. 5. **(A and B)** Live-cell imaging of HEK293T cells co-transfected GRB2-CFP with EV in (A) or RET-KD linked to CRY2oligo-mCherrry in (B) before/after 488 nm blue light induced, n ≥ 3. Scale bar, 10 µm. **(C)** Live-cell imaging of HEK293T cells with co-transfected EGFP-tagged CCDC6-RET or Δ21-27 mutant and GRB2-ECFP, n ≥ 3. Scale bar, 10 µm. **(D)** Live-cell imaging of HEK293T cells with co-transfected RET-KD linked to CRY2oligo-mCherrry and GRB2$^{\Delta SH2}$-CFP before/after 488 nm blue light induced, n ≥ 3. Scale bar, 10 µm. **(E and F)** The LLPS of co-transfection CCDC6-RET with GRB2 or GRB2^{R86K} mutant in HEK293T cells. Live-cell imaging in (E) and the proportion of cells with protein granules in (F), n ≥ 3. Error bars indicate SD from 3 randomly sampled regions with > 200 total cells per region. ***p < 0.001versus GRB2 WT by Student's t test. Scale bar, 10 µm.

Fig S6.

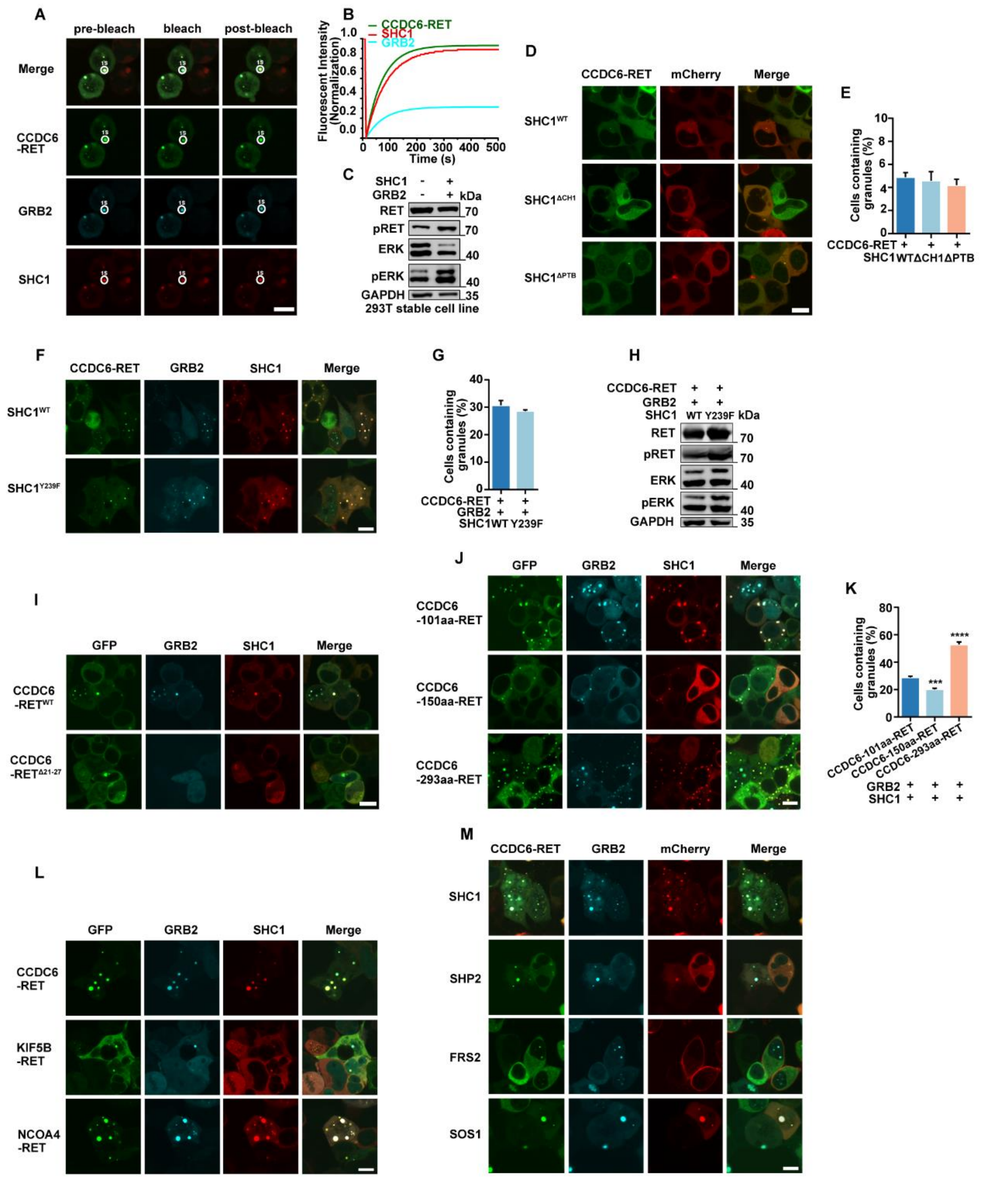

Fig. S6. The RET fusion-GRB2-SHC1 signaling niche is unique for RET-driven Ras/MAPK pathway, related to Fig. 6. **(A and B)** The FRAP of CCDC6-RET-GRB2-SHC1 signaling niche. The FRAP curves in (B) and corresponding droplet at bleached area in (A). Scale bar, 10 µm. **(C)** Western blotting upon HEK293T stable cell lines of CCDC6-RET-EGFP co-transfection GRB2-CFP and SHC1-mCherry. **(D and E)** The LLPS of co-transfection CCDC6-RET with SHC1 WT, ΔCH1 or ΔPTB mutants (tagged by mCherry) in HEK293T cells. Live-cell imaging in (D) and the proportion of cells with protein granules in (E), n ≥ 3. Error bars indicate SD from 3 randomly sampled regions with > 200 total cells per region. ns (non-significant comparison) versus CCDC6-RET by one-way ANOVA. Scale bar, 10 µm. **(F, G and H)** The LLPS of co-transfection CCDC6-

RET-EGFP and GRB2-CFP with SHC1 WT, Y239F mutant (tagged by mCherry) in HEK293T cells. Live-cell imaging in (F), the proportion of cells with protein granules in (G), n ≥ 3, and western blotting in (H). Error bars indicate SD from 3 randomly sampled regions with > 200 total cells per region. ns (non-significant comparison) versus CCDC6-RET by Student's t test. Scale bar, 10 µm. **(I)** Live-cell imaging of HEK293T cells with co-transfected GRB2-ECFP, SHC1-mCherry and EGFP-tagged CCDC6-RET or CCDC6-RET$^{\Delta 21\text{-}27}$ mutant, Scale bar, 10 µm. **(J and K)** The LLPS of co-transfection GRB2-CFP and SHC1-mCherry with CCDC6-101aa-RET-EGFP, CCDC6-150aa-RET-EGFP or CCDC6-293aa-RET-EGFP in HEK293T cells. Live-cell imaging in (J) and the proportion of cells with protein granules in (K), n ≥ 3. Error bars indicate SD from 3 randomly sampled regions with > 200 total cells per region. ****p < 0.0001 and ***p < 0.001 versus CCDC6-RET by Student's t test. Scale bar, 10 µm. **(L)** Live-cell imaging of HEK293T cells with co-transfected GRB2-ECFP, SHC1-mCherry and EGFP-tagged CCDC6-RET, KIF5B-RET and NCOA4-RET, n ≥ 3. Scale bar, 10 µm. **(M)** Live-cell imaging of HEK293T cells with co-transfected GRB2-ECFP, CCDC6-RET-EGFP and mCherry-tagged SHC1, SHP2, FRS2 or SOS1, n ≥ 3. Scale bar, 10 µm.

Fig S7.

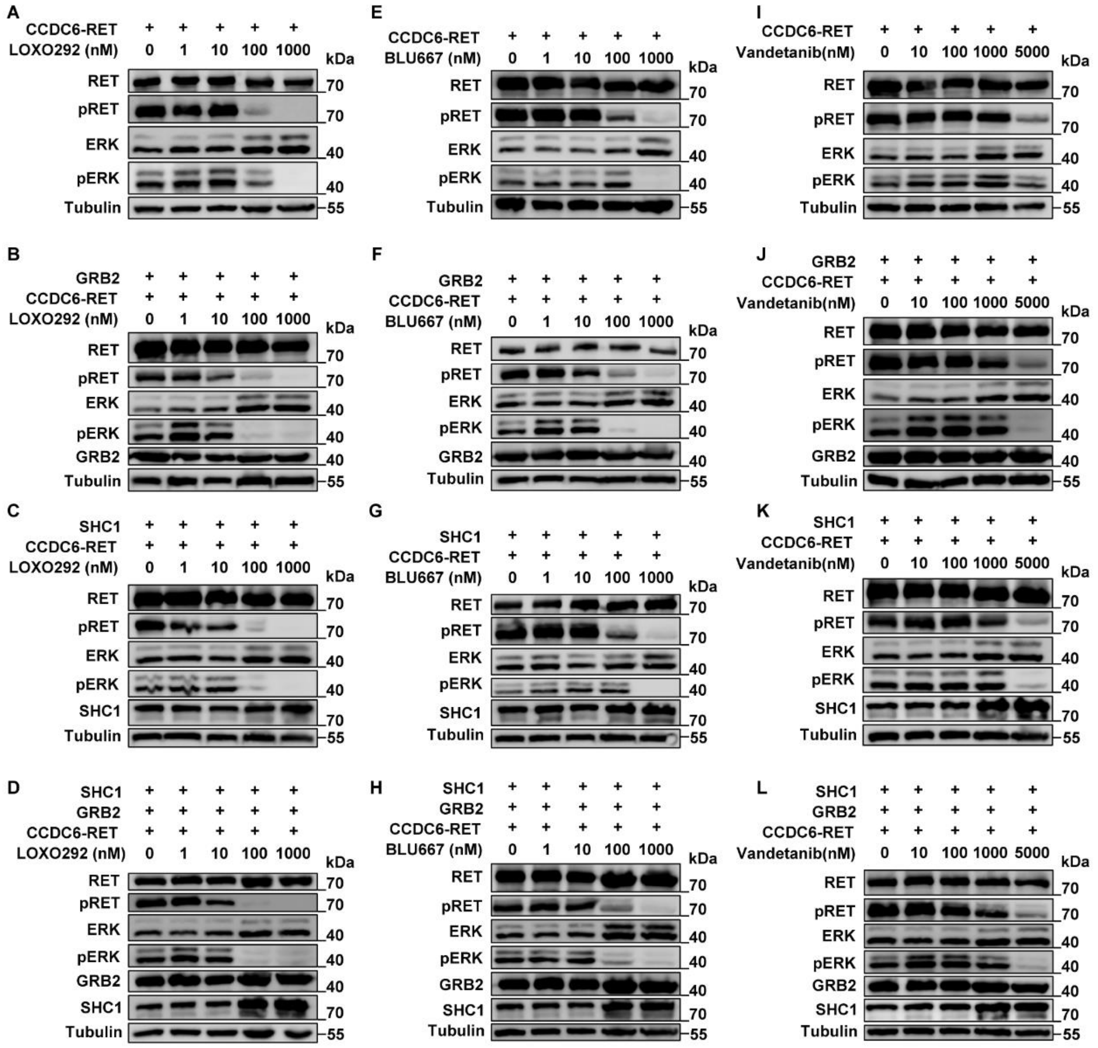

Fig. S7. Effects of RET inhibitors on RET-driven Ras/MAPK signaling pathway. (A, B, C and D) Western blotting upon HEK293T transfection of CCDC6-RET alone in (A), CCDC6-RET along with GRB2 in (B), CCDC6-RET along with SHC1 in (C), and CCDC6-RET along with both GRB2 and SHC1 in (D) treated with LOXO292. (E, F, G and H) Western blotting upon HEK293T transfection of CCDC6-RET alone in (E), CCDC6-RET along with GRB2 in (F), CCDC6-RET along with SHC1 in (G), and CCDC6-RET along with both GRB2 and SHC1 in (H) treated with BLU667. (I, J, K and L) Western blotting upon HEK293T transfection of CCDC6-RET alone in (I), CCDC6-RET along with GRB2 in (J), CCDC6-RET along with SHC1 in (K), and CCDC6-RET along with both GRB2 and SHC1 in (L) treated with vandetanib. The plasmid labels employed in these experiments were as following: CCDC6-RET tagged with EGFP, GRB2 tagged with CFP, and SHC1 tagged with mCherry.

Legends for Movies S1

Movie S1 (separate file). Time-lapse imaging of EGFP-tagged CCDC6 and CCDC6-Δ101aa droplet fusion, related to Fig. 1

(A) A video presentation of CCDC6 protein droplets fusion in cells
(B) A video presentation of CCDC6-Δ101aa protein droplets fusion in cells

SI References

1. O. Shalem *et al.*, Genome-Scale CRISPR-Cas9 Knockout Screening in Human Cells. *Science* **343**, 84-87 (2014).
2. N. E. Sanjana, O. Shalem, F. Zhang, Improved vectors and genome-wide libraries for CRISPR screening. *Nat. Methods* **11**, 783-784 (2014).
3. V. Subbiah *et al.*, Precision Targeted Therapy with BLU-667 for RET-Driven Cancers. *Cancer Discov.* **8**, 836-849 (2018).
4. B. J. Solomon *et al.*, RET solvent front mutations mediate acquired resistance to selective RET inhibition in RET-driven malignancies. *J. Thorac. Oncol.* **15**, 541-549 (2020).
5. F. Carlomagno *et al.*, ZD6474, an orally available inhibitor of KDR tyrosine kinase activity, efficiently blocks oncogenic RET kinases. *Cancer Res.* **62**, 7284-7290 (2002).
